# Fc-engineered antibodies enhance protection against SARS-CoV-2 lung infection and inflammation

**DOI:** 10.1128/mbio.00557-26

**Published:** 2026-04-13

**Authors:** Samantha R. Mackin, Chieh-Yu Liang, Courtney E. Karl, Maksim Kleverov, Mehak Z. Khan, Thendral Selvam, Matthias Mack, Galit Alter, Barbara Guarino, Davide Corti, Michael A. Schmid, Michael S. Diamond

**Affiliations:** 1 Department of Medicine, Washington University School of Medicine 12275 , St. Louis, Missouri, USA; 2 Department of Pathology & Immunology, Washington University School of Medicine 12275 , St. Louis, Missouri, USA; 3 Department of Molecular Microbiology, Washington University School of Medicine 12275 , St. Louis, Missouri, USA; 4 Ragon Institute of MGH, MIT and Harvard, Cambridge, Massachusetts, USA; 5 Department of Nephrology, University Hospital Regensburg 39070, Regensburg, Germany; 6 Vir Biotechnology, Bellinzona, Switzerland; 7 Andrew M. and Jane M. Bursky the Center for Human Immunology and Immunotherapy Programs, Washington University School of Medicine 12275 , St. Louis, Missouri, USA; 8 Center for Vaccines and Immunity to Microbial Pathogens, Washington University School of Medicine 12275 , St. Louis, Missouri, USA; Tsinghua University, Beijing, China

**Keywords:** antibody function, coronavirus, viral pathogenesis, immunotherapy

## Abstract

As the SARS-CoV-2 pandemic progressed, many monoclonal antibodies (mAbs) that neutralized infection against initial strains lost potency against later variants due to the accumulation of mutations in the spike protein. Nonetheless, some mAbs, including the parent of the therapeutically used sotrovimab, S309, remained protective in animals against Omicron variants despite reduced neutralizing potential, with inhibitory activity likely sustained by Fc-mediated effector functions. Here, we identify Fc variants of S309 that confer enhanced protection against SARS-CoV-2 infection in a humanized Fcγ receptor transgenic (Hu-FcγR Tg) mouse model of infection. Versions of S309 that are afucosylated (AFUC) and contain a G236A (GA) mutation in the Fc region showed increased binding to FcγRs IIA, IIIA, and IIIB and enhanced phagocytic activity in cell culture-based assays. Treatment with S309-GA-AFUC resulted in less viral burden, inflammation, and pulmonary ventilatory dysfunction in the lungs of Hu-FcγR Tg mice challenged with SARS-CoV-2 strains compared to the parental S309 mAb or a variant lacking Fc effector functions (S309-GRLR). The enhanced protection in the lung conferred by S309-GA-AFUC required trafficking of CCR2-expressing monocytes to reduce SARS-CoV-2 viral burden and lung injury. Flow cytometry and RNA sequencing analyses showed that compared to the parental S309 mAb, S309-GA-AFUC treatment reduced the inflammatory state and induced a reparative transcriptional signature in monocytes and interstitial macrophages. Overall, our findings demonstrate that Fc engineering to increase antibody binding to activating FcγRs can strengthen effector functions, shape myeloid transcriptional profiles, and enhance protection against SARS-CoV-2 infection *in vivo*.

**IMPORTANCE:**

Although therapeutic antibodies had success in protecting vulnerable individuals from severe COVID-19 during the early stages of the pandemic, many lost effectiveness as SARS-CoV-2 accumulated mutations that compromised neutralizing activity. Our experiments show that antibody protection against SARS-CoV-2 strains can be enhanced by genetically engineering the Fc region or altering its N-linked glycosylation to improve interactions with FcγRs on host immune cells. Modified versions of S309, the parent of the clinically used sotrovimab antibody, more effectively reduce viral burden and inflammation in the lung and shape protective transcriptional responses, which, together, result in improved lung ventilatory function and outcome after SARS-CoV-2 infection. Thus, antibody engineering can serve as a strategy to enhance therapeutic activity against rapidly evolving viruses with the potential to escape neutralization.

## INTRODUCTION

The clinical utility of monoclonal antibodies (mAbs) extends across multiple therapeutic targets in cancer, autoimmunity, and microbial infections, including their recent use against severe acute respiratory syndrome coronavirus 2 (SARS-CoV-2). Several highly neutralizing anti-SARS-CoV-2 mAbs, derived from memory B cells or plasmablasts of immune patients or humanized mice, advanced into humans and demonstrated protection as prophylaxis or early therapeutic activity. Among these, the mAb S309, isolated from a SARS-CoV-infected subject in 2003, targeted a conserved epitope in the receptor binding domain (RBD) of the spike protein and showed robust cross-neutralizing activity against the SARS-CoV-2 Wuhan-1 strain (1).

Sotrovimab, a therapeutic neutralizing antibody developed from the mAb S309, was approved under an emergency use authorization (EUA) and used to reduce hospitalization and mortality among high-risk coronavirus disease 2019 (COVID-19) patients (2, 3). However, over the course of the pandemic, the neutralizing activity of sotrovimab diminished due to the emergence of resistant strains, especially Omicron variant*s* (4, 5). Despite reduced neutralization potency, S309 maintained its ability to protect mice, hamsters, and macaques challenged with Omicron viruses, at least in part due to its Fc-mediated effector functions (6–8). Consistent with these preclinical findings, multiple observational studies in humans demonstrated that sotrovimab maintained effectiveness in preventing severe COVID-19 outcomes during BA.2 and BA.5 infections (9). The antibody-mediated effector functions that could contribute to protection include antibody-dependent cellular phagocytosis (ADCP), antibody-dependent cellular cytotoxicity (ADCC), and antibody-dependent complement deposition (ADCD). ADCP and ADCC are mediated by interactions between the fragment crystallizable (Fc) region of antibodies and Fc gamma receptors (FcγRs) expressed by host cells, including macrophages, monocytes, neutrophils, and natural killer (NK) cells (10, 11). ADCD is mediated by C1q binding to the Fc of antibody-antigen complexes, which activates the classical complement pathway, resulting in phagocytosis or direct lysis of opsonized virions or virally infected cells (12).

The FcγRs responsible for mediating effector functions in humans include FcγRs I (CD64), IIA (CD32A), IIB (CD32B), IIIA (CD16A), and IIIB (CD16B). Activating type I Fc receptors initiate signaling cascades through an immunoreceptor tyrosine-based activating motif (ITAM) contained in their cytoplasmic domain (FcγR IIA) or within an associated common gamma (γ) chain (FcγR I and IIIA); FcγR IIIB, a glycosylphosphatidylinositol (GPI) anchored receptor, does not directly activate γ-chain-associated signaling cascades. To temper activating responses, an inhibitory receptor in humans, FcγR IIB, contains an immunoreceptor tyrosine-based inhibitory motif (ITIM) within its cytoplasmic domain that recruits phosphatases to inhibit ITAM activation cascades. While many immune cells express a combination of activating and inhibitory FcγRs to coordinate a regulated response, B cells only express the inhibitory FcγR IIB, and NK cells only express the activating FcγR IIIA (13). In comparison, type II FcγRs, such as DC-SIGN (CD209) and CD23, have roles in B cell maturation and immunomodulation (14, 15). To study the interactions between human antibodies and their activating human type I FcγRs *in vivo* in a model that more faithfully recapitulates their expression patterns and functions, a transgenic, humanized mouse (Hu-FcγR Tg) was developed. These animals lack murine type I FcγRs and express human type I FcγRs under control of endogenous human regulatory elements and allow for the assessment of human antibody and FcγR functions in a species-matched manner (16).

Engineering of the Fc domain or modification of the N-linked glycans in the CH2 domain can alter the ability of an antibody to bind to specific FcγRs. For example, L234A/L235A/P329G (LALA-PG) or G236R/L328R (GRLR) mutations abolish antibody Fc-FcγR interactions (17). Indeed, the introduction of LALA-PG substitutions into the Fc region of several neutralizing mAbs against SARS-CoV-2 reduced their therapeutic activity in conventional or human angiotensin-converting enzyme 2 (hACE2) transgenic mice (18–21), and antibody-based blocking or depletion studies suggested that monocytes and possibly CD8^+^ T cells contribute to the Fc-dependent protection of intact antibodies (21). In separate studies, Fc mutations G236A (GA) and A330L/I332E (ALIE), which increase FcγR IIA and IIIA binding of casirivimab and imdevimab, two EUA-approved neutralizing anti-SARS-CoV-2 mAbs, enhanced protection against a mouse-adapted SARS-CoV-2 strain when administered as prophylaxis in Hu-FcγR Tg mice (22). In the context of influenza A virus infection, both broadly neutralizing and non-neutralizing antibodies require FcγR engagement to confer optimal protection against morbidity and mortality (23, 24). The Fc mutations GA and ALIE enhanced protection against influenza virus-induced morbidity and mortality in Hu-FcγR Tg mice by promoting dendritic cell maturation and CD8^+^ T cell priming (25). MAbs targeting Ebola virus (EBOV) glycoproteins also required FcγR engagement to confer protection as prophylaxis or therapy (26, 27). Fc mutations G236A/S239D/A330L/I332E (GASD/ALIE) conferred greater protection against EBOV-induced mortality in Hu-FcγR Tg mice (27). An afucosylated (AFUC) anti-EBOV glycoprotein antibody that increased binding to human FcγR IIIA and IIIB conferred superior protection in a lethal challenge model (28).

Here, we aimed to engineer the Fc of S309 to increase its effector functions, augment its protective activity against SARS-CoV-2 beyond its inherent neutralizing capacity, and define the underlying immune-mediated modes of action. We used Hu-FcγR Tg mice to evaluate a panel of S309-Fc variants and identify the mutation(s) that conferred optimal protection against SARS-CoV-2 lung infection and injury. The combination of a GA mutation and afucosylation, which increases binding to FcγRs IIA, IIIA, and IIIB, conferred greater protection against SARS-CoV-2 infection, inflammation, pathology, and disease outcome than the parental S309 mAb. S309-GA-AFUC-mediated protection required trafficking of CCR2^+^ monocytes but not Ly6G^+^ neutrophils for optimal and early virological control. The mitigated lung inflammation associated with S309-GA-AFUC treatment correlated with reduced expression of activation markers on monocytes and macrophages. RNA sequencing showed a shift in the transcriptional profiles of interstitial macrophages from a glycolytic state in S309-treated mice to an oxidative state in those treated with S309-GA-AFUC. Our results in Hu-FcγR Tg mice demonstrate that Fc-engineering to enhance Fc-FcγR interactions can optimize S309-mediated protection against SARS-CoV-2 viral burden, inflammation, and pathology by a CCR2^+^ cell-dependent mechanism.

## RESULTS

### Fc engineering of S309 enhances protection against SARS-CoV-2 B.1.351

Despite waning neutralizing activity, some antibodies that maintain binding to antigenically shifted SARS-CoV-2 spike variants and engage FcRs can retain protective activity (6, 29, 30). To evaluate whether specific Fc modifications could augment protection conferred by S309, a cross-reactive antibody targeting the RBD of SARS-CoV-2, Fc variants were engineered and tested for binding to Wuhan-1 spike-expressing CHO target cells and activation of human FcγR IIA and IIIA-expressing Jurkat cells (Fig. S1). The Fc-modified human mAbs included a control Fc null variant, GRLR (31), GA that enhances FcγR IIA binding (32), AFUC that increases binding to FcγR IIIA (33), and a combined GA-AFUC variant with enhanced binding to both FcγR IIA and IIIA. S309-GA and S309-GA-AFUC showed increased capacity to activate FcγR IIA-expressing Jurkat cells (Fig. S1A), and S309-AFUC and S309-GA-AFUC showed enhanced activation of FcγR IIIA-expressing Jurkat cells (Fig. S1B). These Fc modifications did not alter the ability of S309 to neutralize the SARS-CoV-2 Beta variant B.1.351 (Fig. S1C).

**Fig 1:**
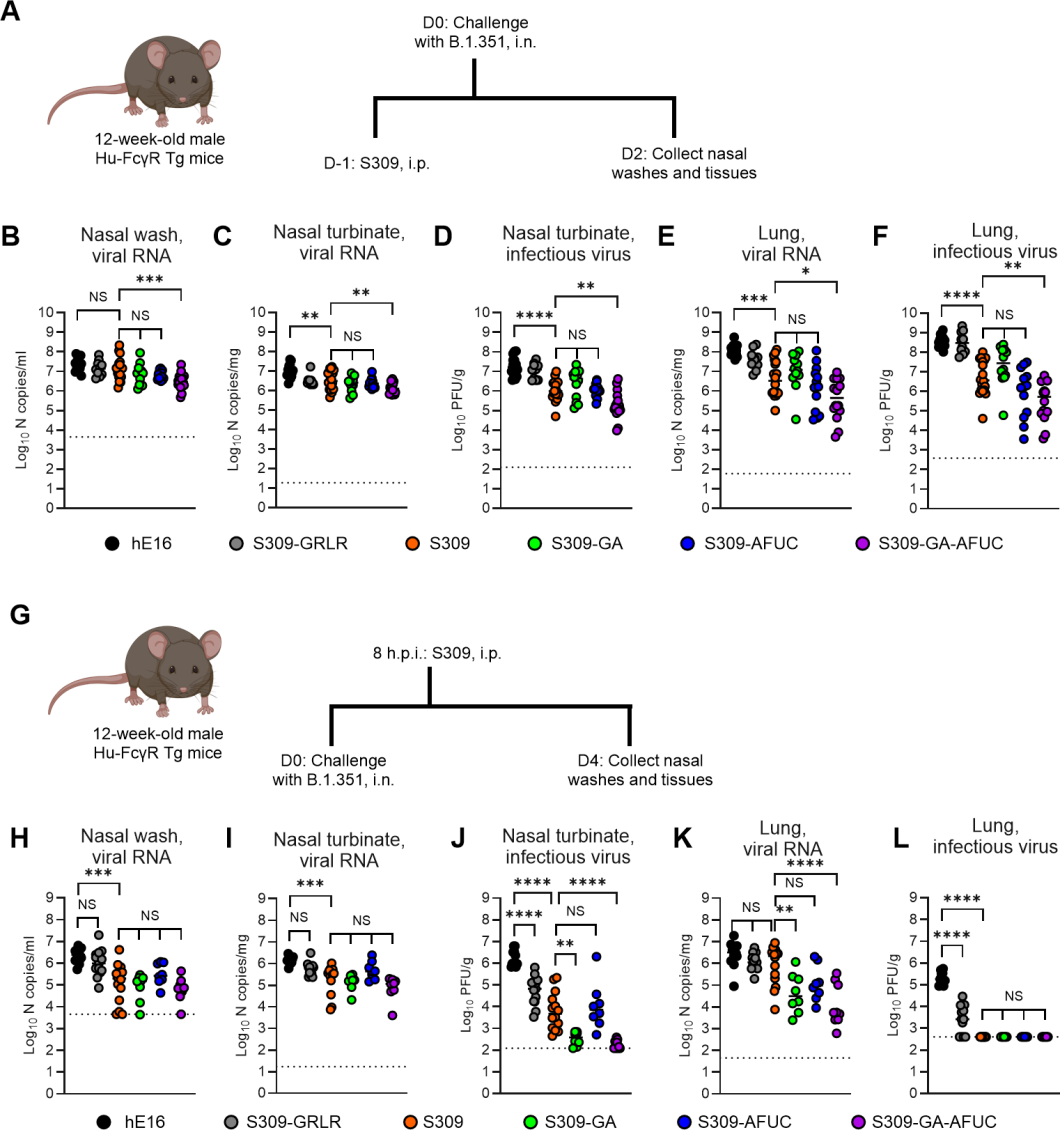
Prophylactic and therapeutic administration of S309 Fc variants against SARS-CoV-2 B.1.351 infection of Hu-FcγR Tg mice. (**A, G**) Scheme of antibody administration, virus challenge, and tissue collection in prophylactic (**A**) and therapeutic (**G**) models. (**B–F and H–L**) Twelve-week-old male Hu-FcγR Tg mice were administered by intraperitoneal injection 3 (**B–F**) or 10 (**H–L**) mg/kg of hE16, S309-GRLR, S309 (parental), S309-GA, S309-AFUC, or S309-GA-AFUC 1 day before (**B–F**) or 8 h after (**H–L**) intranasal challenge with 10^5^ FFU of SARS-CoV-2 B.1.351. At 2 (**B–F**) or 4 (**H–L**) dpi, viral RNA in the nasal washes (**B, H**), nasal turbinates (**C, I**), and lungs (**E, K**) was quantified by qRT-PCR, and infectious virus in the nasal turbinates (**D, J**) and lungs (**F, L**) was measured by plaque assay (bars indicate mean; left to right *n* = 14, 10, 18, 12, 13, and 16 mice per group, three experiments (**B–F**); left to right = 11, 13, 14, 8, 8, and 9 mice per group, three experiments (**H–L**); dotted lines show limit of detection (LOD). Statistical analysis: one-way ANOVA with Tukey’s post-test (NS, not significant; ***P = 0.0005 (**B**); **P = 0.0086, **P = 0.0088 (**C**); ****P < 0.0001, **P = 0.0026 (**D**); *P = 0.0108, ***P = 0.0001 (**E**); ****P < 0.0001, **P = 0.0034 (**F**); ***P = 0.0001 (**H**); ***P = 0.0002 (**I**); **P = 0.0011, ****P < 0.0001 (**J**); **P = 0.0058, ****P < 0.0001 (**K**); and ****P < 0.0001 (**L**).

To assess the impact of Fc-engineering of S309 on protection against SARS-CoV-2 *in vivo*, we used 12-week-old Hu-FcγR Tg mice that lack murine type I Fc receptors and express human type I Fcγ receptors (16). We used these mice, in contrast to conventional C57BL/6 mice, since differences in FcγR expression patterns and their affinity for IgG and its subclasses between species result in distinct functional outcomes of Fc-FcγR interactions that might not reflect human physiology (34, 35). Hu-FcγR Tg mice were challenged with the SARS-CoV-2 strain B.1.351, which contains an N501Y mutation in the RBD that enhances affinity to the murine ACE2 receptor, enabling infection of mice without requiring ectopic human ACE2 expression (36). This B.1.351 variant allowed us to study the effects of S309 variants on SARS-CoV-2 infection in Hu-FcγR Tg mice, which express murine ACE2; this was important because the complex breeding schemes used to generate these mice (16) make it difficult to cross a hACE2 transgene into this background. Because infection of B.1.351 in Hu-FcγR Tg mice caused only mild weight loss (Fig. S2A), we focused the analysis on virological measurements. We first tested the impact of Fc modifications of S309 against SARS-CoV-2 in a prophylaxis model. Twelve-week-old Hu-FcγR Tg mice were treated by intraperitoneal injection with 3 mg/kg of hE16 (anti-West Nile virus isotype control mAb), S309 (parental), S309-GRLR (loss of FcγR binding), S309-GA, S309-AFUC, or S309-GA-AFUC 1 day before challenge with 10^5^ focus-forming units (FFU) of B.1.351. The levels of the different antibodies in the sera and lungs of Hu-FcγR Tg mice were equivalent 3 days post-administration, establishing that differences in phenotypes were not due to variation in mAb bioavailability (Fig. S3). At 2 days post-infection (dpi), we measured viral burden in the nasal washes, nasal turbinates, and lungs (Fig. 1A). S309 treatment had little substantive impact on SARS-CoV-2 viral RNA levels in the nasal washes (Fig. 1B). However, it conferred greater protection in the nasal turbinates and lungs (lower viral RNA and infectious virus levels) than the isotype control mAb (Fig. 1C through F). In the nasal washes, nasal turbinates, and lungs, we observed reduced SARS-CoV-2 viral RNA or infectious virus in animals treated with S309-GA-AFUC compared to those receiving parental S309, S309-GA, or S309-AFUC (Fig. 1B through F). These data suggest that S309 prophylaxis against SARS-CoV-2 respiratory tract infection in Hu-FcγR Tg mice can be improved by introducing modifications that enhance binding to FcγRs IIA and IIIA, with enhanced protection achieved through the combined effects of both receptor interactions.

We next assessed protection by the S309 Fc variants in the context of post-exposure therapy. Eight hours after intranasal challenge with 10^5^ FFU of B.1.351, mice were administered by intraperitoneal injection of 10 mg/kg of hE16, S309 (parental), S309-GRLR, S309-GA, S309-AFUC, or S309-GA-AFUC. At 4 dpi, nasal washes, nasal turbinates, and lungs were collected for viral burden analysis (Fig. 1G). At the dose tested, parental S309 mAb treatment diminished SARS-CoV-2 B.1.351 RNA levels in the nasal washes and turbinates but did not reduce infection in the lungs compared to animals receiving isotype control mAb (Fig. 1H, I, and K). However, infectious virus titers in the nasal turbinates and lungs were reduced in animals receiving S309-GRLR and parental S309 compared to the isotype control mAb (Fig. 1J and L). In samples from the upper respiratory tract, statistically significant differences in SARS-CoV-2 viral RNA were not observed among groups receiving S309 endowed with different FcγR-binding activities (Fig. 1H and I). However, treatment with S309-GA and S309-GA-AFUC resulted in less infectious virus in the nasal turbinates than animals receiving parental S309, suggesting that the modified mAbs improve viral clearance (Fig. 1J). In the lung, viral RNA was also lower in the S309-GA- and -GA-AFUC-treated animals than in those receiving parental S309 mAb. Nonetheless, by 4 dpi, infectious virus in the lung was undetectable in most S309 mAb-treated groups (Fig. 1L). Collectively, these data suggest that the therapeutic activity of S309 against SARS-CoV-2 in the upper and lower respiratory tract of Hu-FcγR Tg mice is enhanced by Fc engineering that augments binding to FcγRs IIA, IIIA, and/or IIIB.

To elucidate the possible mechanisms of enhanced protection, we profiled parental S309, S309-GRLR, and S309-GA-AFUC to evaluate the impact of Fc modifications on human FcγR binding and *in vitro*-based effector functions (37). We first incubated B.1.351 spike-coated beads with the S309 mAbs and then tested them for binding to individual human FcγRs (FcγR I, IIA [H131 and R131 allotypes], IIB, IIIA, and IIIB) in solution (Fig. S4A). As expected, S309-GRLR did not bind any FcγRs. In comparison, S309-GA-AFUC showed increased binding to FcγR I, IIA, IIIA, and IIIB compared to parental S309. In cellular assays, S309-GA-AFUC demonstrated enhanced antibody-dependent neutrophil phagocytosis (ADNP) activity and increased NK cell activation (ADNKA, measured by CD107a expression) compared to the parental S309 following exposure to B.1.351 spike-coated beads (Fig. S4B and C). Unexpectedly, parental S309 promoted ADCD on B.1.351 spike-coated beads more than S309-GA-AFUC did (Fig. S4D). S309-GA-AFUC and parental S309 induced similar levels of ADCP of B.1.351 spike-coated beads in cultured THP-1 monocytic cells (Fig. S4E). S309-GRLR did not induce phagocytosis or cell activation above background in any assay. These *in vitro* studies suggest that specific Fc effector function activities are enhanced by S309-GA-AFUC, such as ADNP and ADNKA, which might contribute to the greater protection *in vivo*.

### Fc-engineered S309 mAb requires CCR2^+^ cells for optimal virological protection against SARS-CoV-2 B.1.351

**Fig 2:**
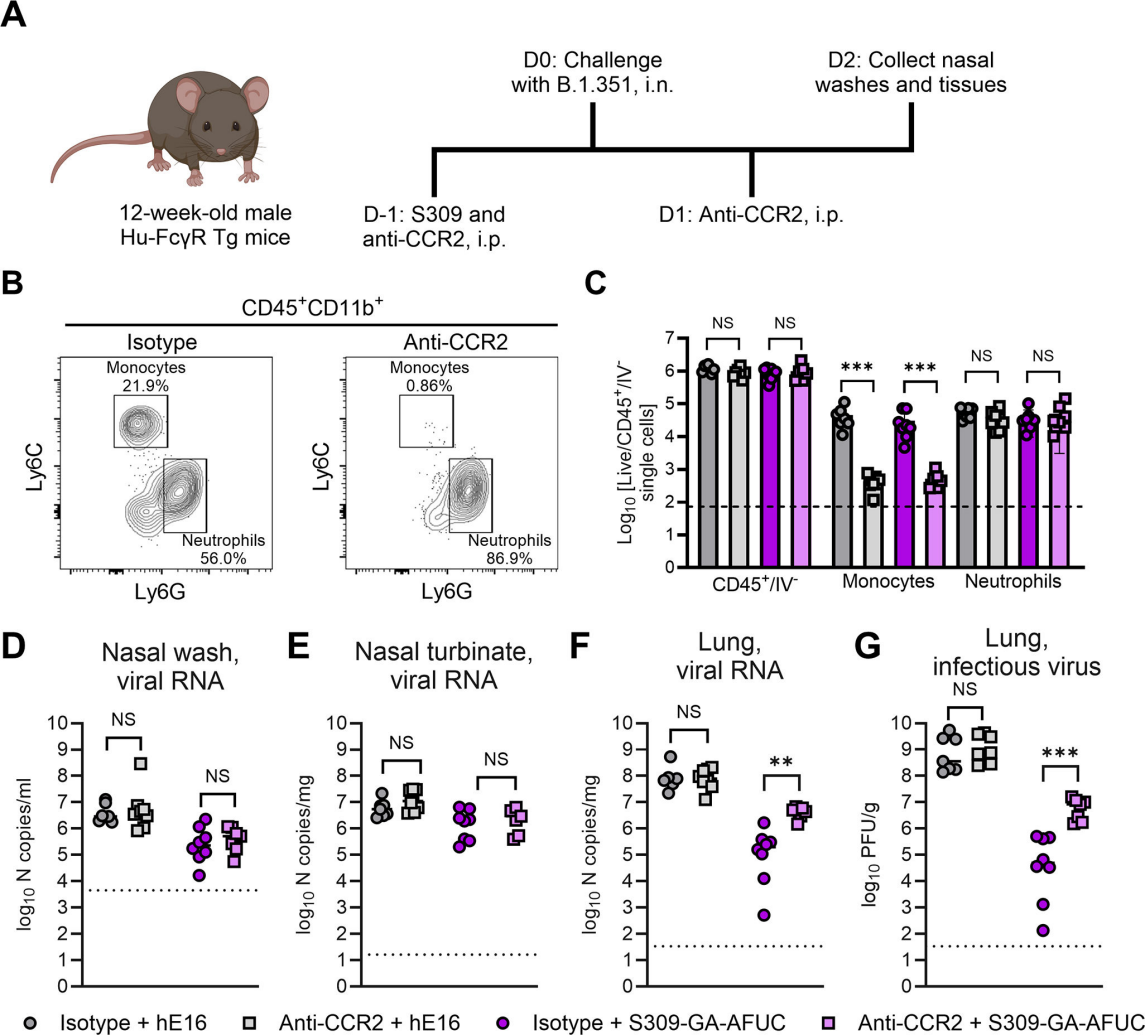
Anti-CCR2 mAb treatment impairs the protective activity of S309-GA-AFUC against B.1.351 infection. (**A**) Scheme of antibody administration, virus challenge, and tissue collection. (**B**) Representative flow cytometry plots showing depletion of monocytes in the lungs at 4 dpi, with numbers showing the cell population as a percentage of CD45^+^CD11b^+^ cells. (**C**) Flow cytometric analysis of monocytes and neutrophils in the lungs of mice receiving anti-CCR2 or isotype antibody at 4 dpi. Dotted lines show the limit of detection (LOD). (**D–G**) Twelve-week-old male Hu-FcγR Tg mice were administered 3 mg/kg of hE16 or S309-GA-AFUC by intraperitoneal injection 1 day before intranasal challenge with 10^5^ FFU of SARS-CoV-2 B.1.351. Mice were also administered 50 μg of anti-CCR2 (MC-21) or rat IgG2b isotype control by intraperitoneal injection 1 day before and after challenge. At 2 dpi, viral RNA in the nasal washes (**D**), nasal turbinates (**E**), and lungs (**F**) was quantified, and infectious virus in the lungs (**G**) was determined (bars indicate mean; left to right *n* = 7, 8, 8, and 7 mice per group, two experiments, dotted lines show LOD. Statistical analysis: one-way ANOVA with Tukey’s post-test (NS, not significant; ***P = 0.0003, ***P = 0.0003 (**C**); **P = 0.0023 (**F**); ***P = 0.0007 [**G**]). (**C**–**G**) Colored symbols indicating antibody treatment conditions are shown at the bottom of the Figure.

We next evaluated which FcγR-expressing immune cells were required for the greater protection observed with S309-GA-AFUC. Immune cell subsets in Hu-FcγR Tg mice recapitulate the expression patterns seen in humans (16, 22). We hypothesized that monocytes and/or other myeloid cells might contribute based on their expression of activating FcγRs (Fig. S5) and ability to migrate into the lung upon SARS-CoV-2 infection (38). To address this question, we administered 50 μg of anti-CCR2 (MC-21), a mAb that inhibits monocyte trafficking into tissues (39), by intraperitoneal injection 1 day before and after challenge with 10^5^ FFU of B.1.351 (Fig. 2A). Mice were also treated with 3 mg/kg of hE16 or S309-GA-AFUC by intraperitoneal injection 1 day before challenge, as we did in our prophylaxis studies (Fig. 1A through F). At 2 dpi, nasal washes, nasal turbinates, and lungs were collected. Flow cytometric analysis confirmed that anti-CCR2 treatment specifically reduced the number of Ly6C^hi^ monocytes in the lung (Fig. 2B and C). In the nasal washes and turbinates, SARS-CoV-2 viral RNA levels were similar between isotype- and anti-CCR2-treated mice (Fig. 2D through E). Anti-CCR2 also had no impact on viral burden in the lungs of animals receiving isotype control mAb but resulted in less effective control of SARS-CoV-2 infection (both viral RNA and infectious virus) in the lungs of S309-GA-AFUC-treated mice (Fig. 2F and G). Thus, S309-GA-AFUC treatment requires CCR2^+^ monocytes or monocyte-derived cells for optimal protection at an early time point (2 dpi) against SARS-CoV-2 infection in the lungs of Hu-FcγR Tg mice.

### Fc engineering enhances S309-mediated protection against lung injury caused by a mouse-adapted SARS-CoV-2 strain

**Fig 3:**
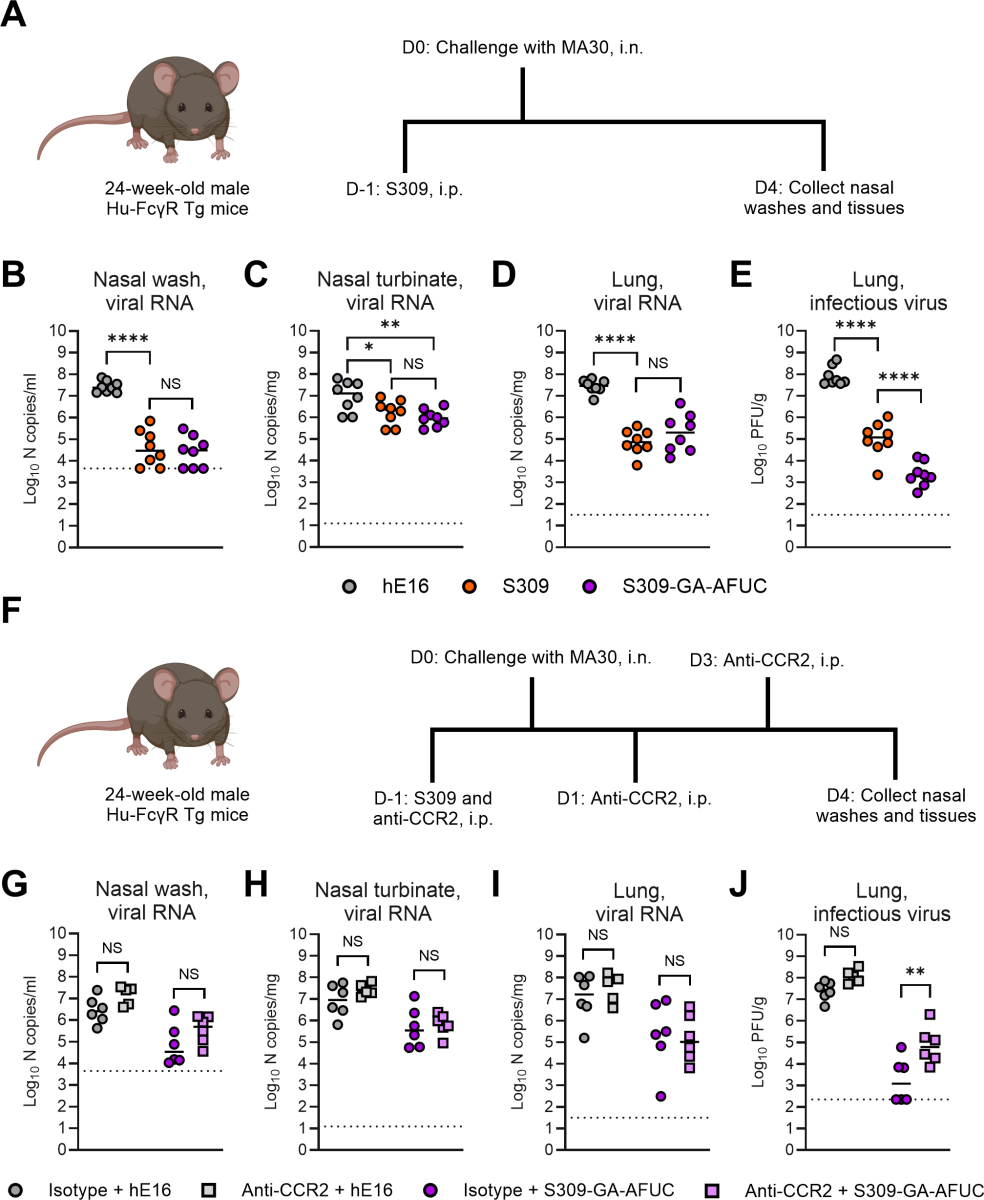
Prophylaxis of S309 Fc variants, treatment with anti-CCR2 mAb, and SARS-CoV-2 MA30 infection of Hu-FcγR Tg mice. (**A**) Scheme of antibody administration, virus challenge, and tissue collection. (**B–E**) Twenty-four-week-old male Hu-FcγR Tg mice were administered by intraperitoneal injection 6 mg/kg of hE16, S309 (parental), or S309-GA-AFUC 1 day before intranasal challenge with 6 × 10^5^ FFU of SARS-CoV-2 MA30. At 4 dpi, viral RNA in the nasal washes (**B**), nasal turbinates (**C**), and lungs (**D**) was quantified, and infectious virus in the lungs (**E**) was measured (bars indicate mean; *n* = 8 mice per group, two experiments (**B–E**), dotted lines show LOD). (**F**) Scheme of antibody administration, virus challenge, and tissue collection. (**G–J**) Twenty-four-week-old male Hu-FcγR Tg mice were administered by intraperitoneal injection 6 mg/kg of hE16, S309 (parental), or S309-GA-AFUC 1 day before intranasal challenge with 6 × 10^5^ FFU of SARS-CoV-2 MA30. Mice were also administered 50 μg of anti-CCR2 (MC-21) or rat IgG2b isotype control by intraperitoneal injection on D-1, +1, and +3 relative to challenge. At 4 dpi, viral RNA in the nasal washes (**G**), nasal turbinates (**H**), and lungs (**I**) was quantified, and infectious virus in the lungs (**J**) was measured (bars indicate mean; left to right *n* = 6, 5, 6, and 6 mice per group, two experiments (**G–J**), dotted lines show LOD). Statistical analysis: one-way ANOVA with Tukey’s post-test (NS, not significant; ****P < 0.0001 (**B**); *P = 0.0419, **P = 0.0058 (**C**); ****P < 0.0001 (**D-E**); **P = 0.0068 (**J**)).

Our studies with SARS-CoV-2 B.1.351 were limited because infected Hu-FcγR Tg mice did not develop severe clinical disease (Fig. S2A). To examine whether the improved protection conferred by an Fc-engineered mAb extends beyond a virological phenotype, we repeated studies using older animals (24-week-old) and the mouse-adapted virus, MA30, which results in severe lung infection and disease in mice (40, 41). MA30 resulted in similar weight loss in K18-hACE2 mice and Hu-FcγR Tg mice (Fig. S2B). We also confirmed that neutralizing activity against MA30 was comparable between parental S309 and S309-GA-AFUC (Fig. S1D). Hu-FcγR Tg mice were treated by intraperitoneal injection with hE16 (isotype control), S309 (parental), or S309-GA-AFUC 1 day before challenge with 6× 10^5^ FFU of MA30. At 4 dpi, nasal washes, nasal turbinates, and lungs were collected, and viral burden was measured (Fig. 3A). While parental S309 mAb reduced viral RNA levels in the nasal washes, turbinates, and lungs compared to animals receiving isotype control mAb (Fig. 3B through D), S309-GA-AFUC better limited the accumulation of infectious virus in the lungs (Fig. 3E). We also confirmed that CCR2-expressing cells contribute to S309-GA-AFUC-mediated protection during MA30 infection. We administered 50 µg of anti-CCR2 to Hu-FcγR Tg mice by intraperitoneal injection at D-1, +1, and +3 relative to challenge with MA30 (Fig. 3F). Mice were also treated with hE16 or S309-GA-AFUC by intraperitoneal injection 1 day before challenge. At 4 dpi, nasal washes, nasal turbinates, and lungs were collected. Viral RNA levels in the nasal washes, turbinates, and lungs were similar between isotype- and anti-CCR2-treated mice (Fig. 3G through I). However, in the lungs of S309-GA-AFUC-treated mice, CCR2 blockade resulted in reduced control of infectious SARS-CoV-2 (Fig. 3J).

Because our cell culture-based Fc effector assay results suggested superior ADNP by Fc-modified S309 (Fig. S4B), we evaluated the contribution of neutrophils to S309-GA-AFUC-mediated protection by administering 100 μg of anti-Ly6G (1A8-CP129) to Hu-FcγR Tg mice at D-1, +1, and +3 relative to challenge with MA30 (Fig. S6A). Mice were treated with hE16 or S309-GA-AFUC by intraperitoneal injection 1 day before challenge. At 4 dpi, nasal turbinates and lungs were collected. Flow cytometric analysis confirmed that anti-Ly6G treatment resulted in marked reductions in neutrophil populations in the lung without effects on other immune cells, including the numbers and activation states of resident F4/80^+^ macrophages and Ly6C^hi^ monocytes (Fig. S6B through E). Anti-Ly6G treatment and neutrophil depletion did not affect S309-GA-AFUC-mediated protection against MA30 infection in the nasal turbinates or lungs (Fig. S6F through I). Thus, S309-GA-AFUC improved virological protection compared to parental S309 after MA30 challenge, and CCR2^+^ monocytes or monocyte-derived cells, but not neutrophils, contributed to this result.

**Fig 4:**
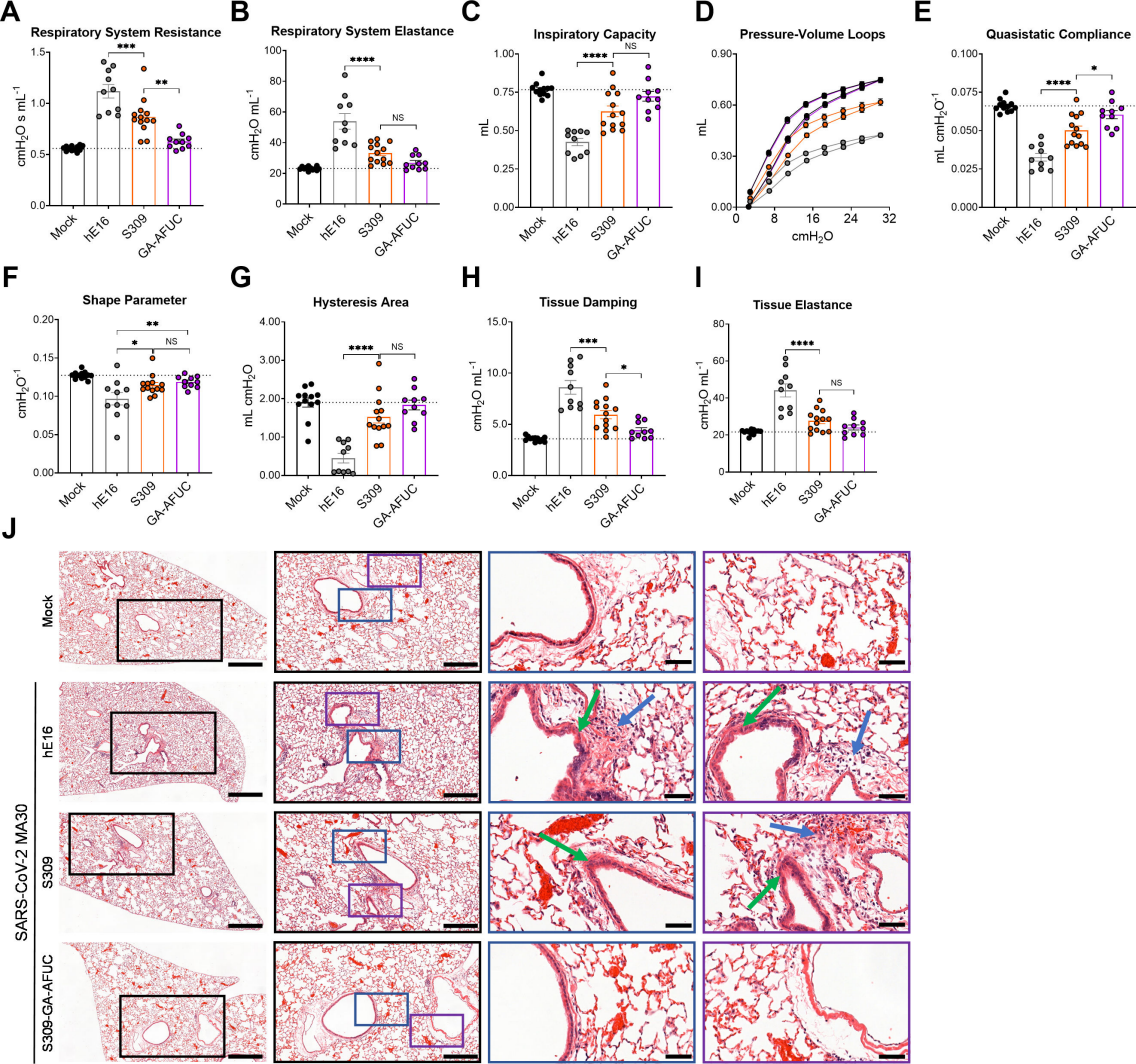
Effects of Fc-engineering of S309 on ventilatory function and pathology caused by MA30 infection in Hu-FcγR Tg mice. Twenty-four-week-old male Hu-FcγR Tg mice were administered hE16, S309, or S309-GA-AFUC by intraperitoneal injection 1 day before intranasal challenge with 6 × 10^5^ FFU of SARS-CoV-2 MA30. Mock-infected animals were inoculated with sterile PBS. At 7 dpi, parameters of respiratory mechanics were measured including resistance (**A**), elastance (**B**), inspiratory capacity (**C**), pressure-volume loops (**D**), quasistatic compliance (**E**), shape parameter (**F**), hysteresis area (**G**), tissue damping (**H**), and tissue elastance (**I**) (bars indicate mean ± standard error of the mean (SEM); left to right *n* = 12, 10, 13, and 10 mice per group, three experiments (**A–I**), dotted lines show mean of mock-infected animals. Statistical analysis: one-way ANOVA with Tukey’s post-test (NS, not significant; ***P = 0.0009, **P = 0.0014 (**A**); ****P < 0.0001 (**B-C**); ****P < 0.0001, *P < 0.0212 (**E**); *P = 0.0311, **P = 0.0086 (**F**); ****P < 0.0001 (**G**); ***P = 0.0001, *P < 0.0427 (**H**); ****P < 0.0001 [**I**]). (**J**). Stained lung sections from 24-week-old male Hu-FcγR Tg mice administered hE16, S309, or S309-GA-AFUC 1 day before intranasal challenge with SARS-CoV-2 MA30 at 7 dpi. Mock-infected animals were inoculated with sterile PBS. From left to right, images show low- (scale bars, 500 μm), medium- (middle; scale bars, 250 μm), and two high-power magnifications (scale bars, 50 μm) of the lungs. Boxed insets indicate the areas of magnification. Green arrows show bronchial wall thickening; blue arrows show immune cell infiltration. Images are representative of *n* = 6 per group.

We also evaluated the functional protection conferred by S309-GA-AFUC against the more pathogenic MA30 strain by measuring pulmonary mechanics using a lung ventilator. Twenty-four-week-old Hu-FcγR Tg mice were treated by intraperitoneal injection with hE16 (isotype control), S309 (parental), or S309-GA-AFUC 1 day before challenge with MA30, and at 7 dpi, lung dynamics were measured. Compared to mock-infected controls, isotype mAb control-treated and infected animals showed abnormal lung mechanics with increased resistance and elastance and reduced inspiratory capacity (Fig. 4A through C). These measurements were associated with a downward deflection of pressure-volume loops (Fig. 4D), decreased elasticity (Fig. 4E), a lower shape parameter, *K* (which describes the curvature of the upper portion of the pressure-volume loop) (Fig. 4F), and decreased hysteresis (Fig. 4G). Isotype control-treated MA30-infected mice also showed increased tissue damping (Fig. 4H) and tissue elastance (Fig. 4I). These ventilatory measurements indicate that MA30 infection causes increased airway resistance and stiffening and decreased lung compliance and distensibility, which is consistent with disease seen in humans (42, 43). Although treatment with S309 improved several respiratory parameters, mice administered S309-GA-AFUC had better pulmonary mechanics than those given parental S309, including respiratory system resistance (Fig. 4A), quasistatic compliance (Fig. 4E), and tissue damping (Fig. 4H).

We next assessed the effects of S309 mAbs on MA30-induced lung pathology. Hu-FcγR Tg mice were treated by intraperitoneal injection with hE16 (isotype control), parental S309, or S309-GA-AFUC 1 day before challenge with MA30. Analysis of lung sections from isotype control-treated MA30-infected mice at 7 dpi revealed evidence of immune cells in the airways, alveolar wall thickening, and mild lung consolidation compared to mock-infected animals. Mice treated with parental S309 exhibited reduced inflammation relative to isotype-treated mice, although immune cells in the airways and bronchial wall thickening persisted. In comparison, S309-GA-AFUC treatment showed lung histology that resembled mock-infected, naive mice (Fig. 4J).

**Fig 5:**
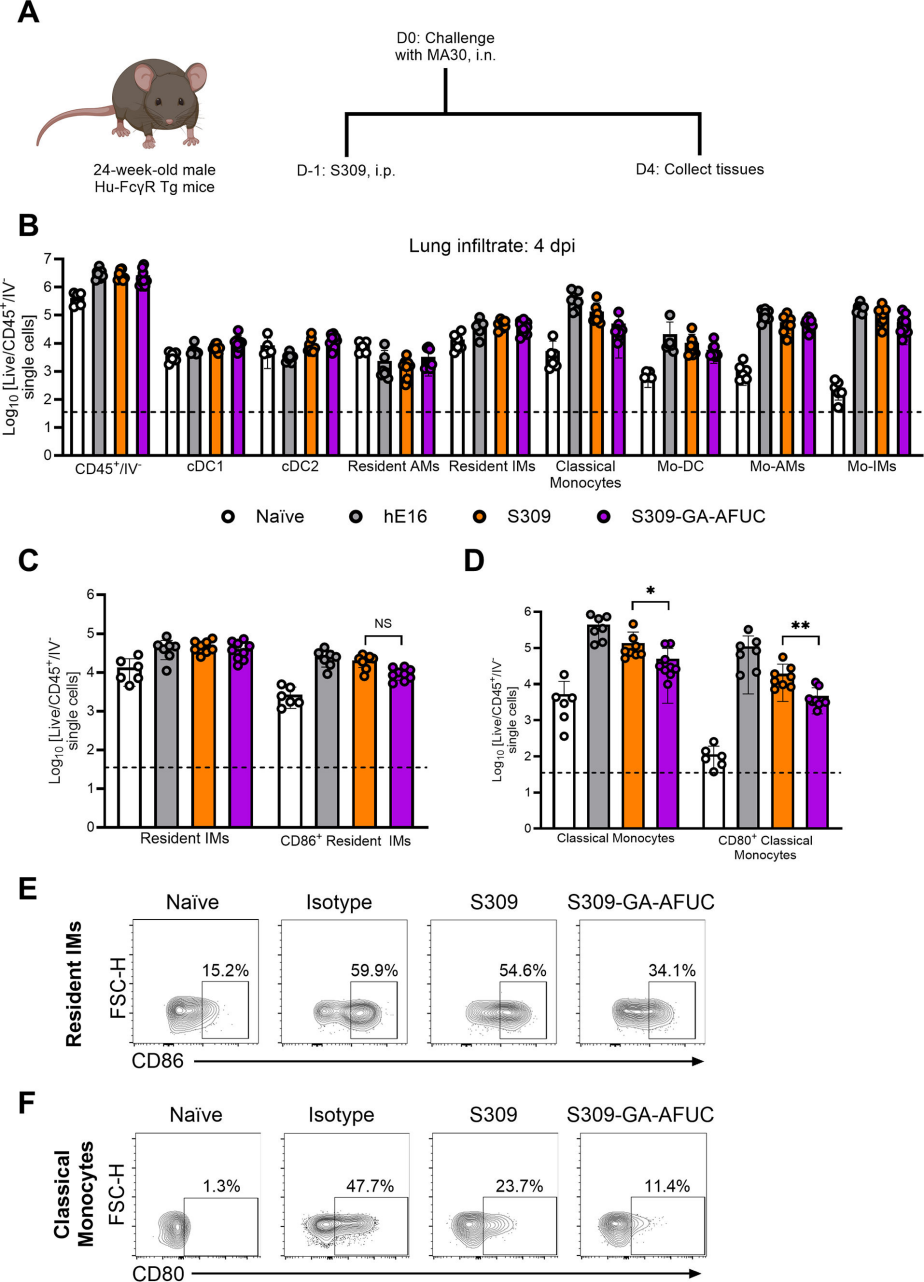
Fc-engineering of S309 decreases the activation of myeloid cells in the lungs of Hu-FcγR Tg mice after MA30 infection. (**A**) Scheme of antibody administration, virus challenge, and tissue collection. (**B–F**) Twenty-four-week-old male Hu-FcγR Tg mice were administered by intraperitoneal injection 6 mg/kg of hE16, S309 (parental), or S309-GA-AFUC 1 day before intranasal challenge with 6 × 10^5^ FFU of SARS-CoV-2 MA30. (**B–D**) Flow cytometric analysis of lung tissues at 4 dpi. (**E**) Representative flow cytometry plots of CD86 expression on resident interstitial macrophages isolated from the lung tissues of the indicated antibody treatment groups. (**F**) Representative flow cytometry plots of CD80 expression on Ly6C^hi^ monocytes isolated from the lung tissues of the indicated antibody treatment groups. Gating scheme in Fig. S7 (bars indicate mean ± SEM; from left to right *n* = 6, 7, 8, and 9 mice per group, three experiments, dotted lines show LOD). Statistical analysis: between S309 and S309-GA-AFUC, two-tailed Mann-Whitney test; **P = 0.005 (**C**); *P = 0.026, **P = 0.0016 (**D**).

To characterize the immune cells that accumulated in the lungs after MA30 infection, we performed spectral flow cytometry. Hu-FcγR Tg mice were treated by intraperitoneal injection with hE16 (isotype control), parental S309, or S309-GA-AFUC 1 day before challenge with MA30 (Fig. 5A). Analysis of lung tissues at 4 dpi (Fig. 5B; Fig. S7A and B) showed a decrease in the number of activated CD80^+^ monocytes (CD11b^+^Ly6C^+^Ly6G^-^MHC-II^-^) and CD86^+^-resident interstitial macrophages (CX3CR1^+^CD88^+^MHC-II^+^Ly6C^-^DC-SIGN^-^) in S309-GA-AFUC-treated animals compared to those receiving parental S309 (Fig. 5C through F).

**Fig 6:**
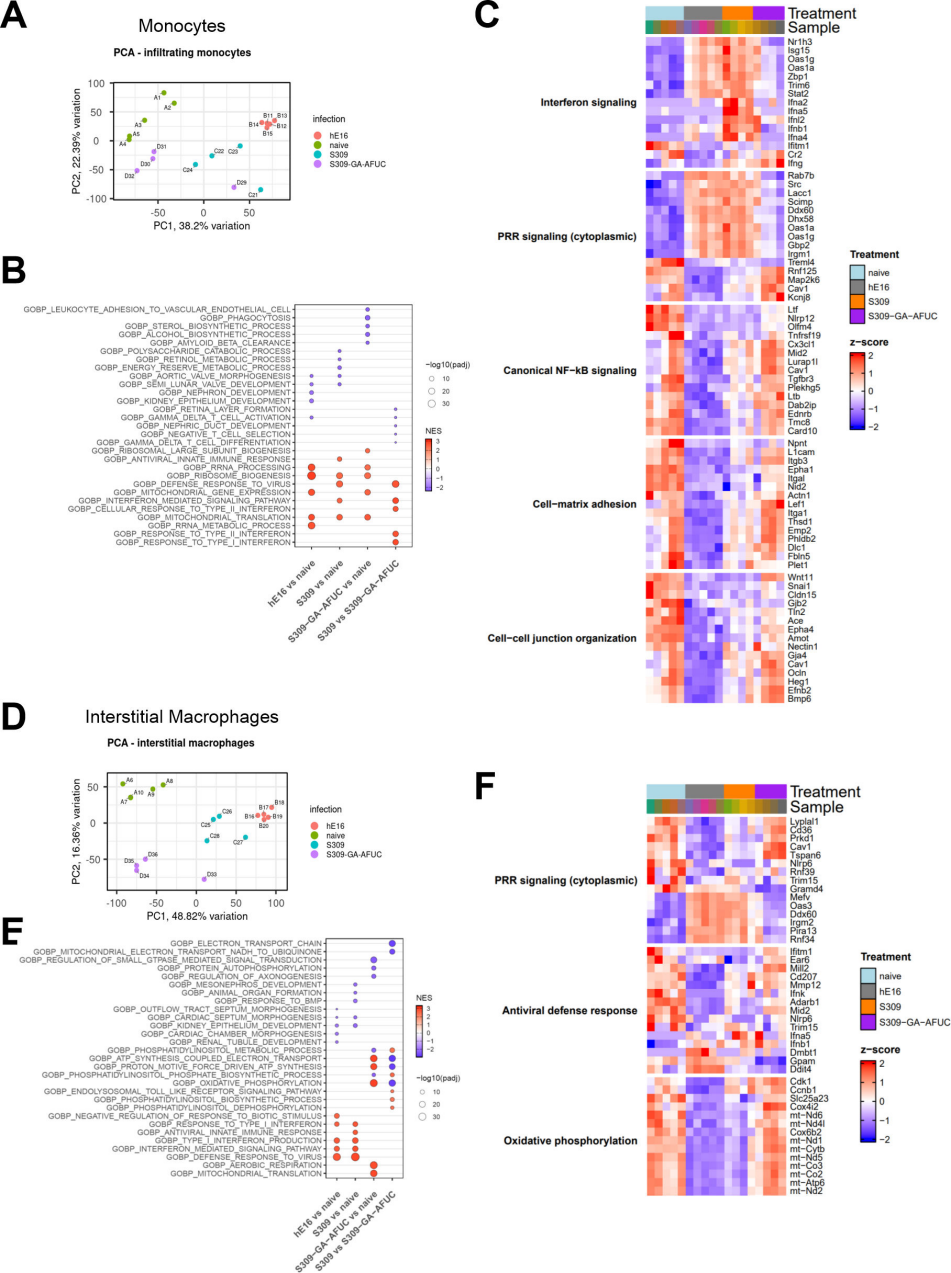
Fc-engineering of S309 alters the transcriptional profile of monocytes and interstitial macrophages in the lungs of Hu-FcγR Tg mice after MA30 infection. RNA sequencing analysis from sorted Ly6G^-^Ly6C^+^CD115^+^ monocytes (**A–C**) or MHCII^+^CD88^+^CX3CR1^+^ interstitial macrophages (**D–F**) isolated from the lungs of 24-week-old male Hu-FcγR Tg mice that were uninfected (naïve) or 3 dpi following intranasal challenge with 6 × 10^5^ FFU of SARS-CoV-2 MA30 and treated with 6 mg/kg of hE16, S309 (parental), or S309-GA-AFUC 1 day before challenge. (**A, D**) Principal component analysis of transcriptional profiles of monocytes (**A**) and interstitial macrophages (**D**). (**B, E**) Gene Ontology (GO) enrichment analysis of differentially expressed pathways in monocytes (**B**) and interstitial macrophages (**E**). (**C, F**) Heatmaps of unbiased, hierarchical clustering of selected genes involved in IFN signaling, cytoplasmic pathogen receptor signaling, NF-κB signaling, cell-matrix adhesion, and cell-cell junction organization of all treatment groups in monocytes (**C**) and interstitial macrophages (**F**). For GO analysis, circle size corresponds to the −log₁₀ adjusted *P*-value, and color indicates normalized enrichment score (NES).

To further define the impact of Fc modifications of S309 mAb on lung inflammation, we performed bulk RNA sequencing on sorted myeloid cell populations at 3 dpi, a time point when antibody-mediated differences in viral burden are beginning to be observed. Hu-FcγR Tg mice were treated with hE16 (isotype control), parental S309, or S309-GA-AFUC 1 day before challenge with MA30. Sorted lung monocytes (Ly6G^-^Ly6C^+^CD115^+^) and resident interstitial macrophages (CD88^+^MHC-II^+^CX3CR1^+^) were subjected to RNA sequencing (Fig. S8). The transcriptional signature of lung monocytes and interstitial macrophages from S309-GA-AFUC-treated animals aligned more closely to mock-infected naive animals, whereas those from parental S309-treated animals were more like isotype-treated MA30-infected mice (Fig. 6A and D). Gene ontology analysis revealed an upregulation of antiviral genes after MA30 infection in isotype control and parental S309-treated monocytes and interstitial macrophages (Fig. 6B and E). In comparison, S309-GA-AFUC treatment showed diminished antiviral signatures in monocytes and an enrichment of transcripts associated with oxidative phosphorylation, electron transport chain, and mitochondrial translation, consistent with a shift toward a metabolically reparative state. A heat map analysis showed that compared to cells from uninfected animals, SARS-CoV-2 infection resulted in marked upregulation of interferon (IFN)-stimulated genes (e.g., *Isg15*, *Oas1a*, *Ifna2*, and *Ifnb1*) and pattern recognition receptor signaling (e.g., *Rab7b*, *Src*, *Irgm1*, and *TremI4*) in monocytes from hE16- and S309-treated animals, indicative of innate immune activation and an antiviral response (Fig. 6C). Monocytes isolated from SARS-CoV-2-infected and S309-GA-AFUC-treated animals exhibited an attenuated IFN response and increased expression of genes involved in membrane remodeling and cell adhesion (e.g., *L1cam*, *Itgb3*, *Itga1*, and *Epha1*), and with reparative or cell-cell junction organization (e.g., *Wnt11*, *Snai1*, and *Cldn15*).

RNA sequencing of sorted interstitial macrophages at 3 dpi similarly revealed induction of genes involved in pathogen recognition signaling (e.g., *Ddx60*, *Oas3*, *Irgm2*, and *Mefv*) and type I IFN responses (e.g., *Ifna5*, *Ifnb1*, *Ifnk*, and *Ifitm1*) in hE16- and S309-treated animals (Fig. 6F). Expression of these antiviral programs was lower in S309-GA-AFUC-treated animals, whereas genes associated with oxidative phosphorylation (e.g., *Cox4i2*, *mt-Co3*, *mt-Nd5*, and *mt-Atp6*) were upregulated. Collectively, these data suggest that Fc-engineering of S309 results in more rapid viral clearance, which results in reprogramming of monocytes and interstitial macrophages from antiviral and inflammatory phenotypes to ones characterized by immune regulation, metabolic reprogramming, and repair. Together, the ventilation, histopathology, flow cytometry, and RNA sequencing data suggest that S309-GA-AFUC can better control infection in the lungs soon after virus exposure, resulting in reduced pathological inflammation compared to isotype control and parental S309 mAb treatments.

## DISCUSSION

Neutralization potency has historically been considered a key correlate of protection for vaccines and antibody therapeutics against infections. However, antibodies with limited or no neutralizing activity can have substantial inhibitory activity *in vivo*, suggesting alternative protective mechanisms, including Fc-mediated effector functions. The role of Fc-FcγR interactions in antibody protection has been demonstrated in several virus models, and Fc engineering to enhance FcγR engagement can improve therapeutic efficacy (28, 44, 45). Despite these advances, the underlying mechanisms responsible for the enhanced protection conferred by specific Fc modifications have not been fully elucidated and may vary depending on the virus, tissue location, and cause of pathogenesis. In this study, we identified the combination of a GA mutation and afucosylation (GA-AFUC) as modifications that enhance protection against SARS-CoV-2 in transgenic mice expressing human FcγRs.

Our immune cell depletion results suggested that CCR2-expressing myeloid cells contribute to the enhanced protection of S309-GA-AFUC by promoting accelerated viral clearance, possibly through antibody-dependent phagocytosis. Although S309-GA-AFUC did not enhance ADCP activity in cell culture assays compared to parental S309 in THP-1 cells, these results may not fully reflect *in vivo* activity: (i) THP-1 cells are an undifferentiated monocytic cell line and do not fully recapitulate the FcγR expression patterns or functional capacity of tissue resident phagocytes in the lung, and (ii) although bead-based ADCP assays model the uptake of antibody-opsonized virions, antibody-mediated phagocytosis *in vivo* includes clearance of infected cells that display viral antigen on their surface. Therefore, the findings in *ex vivo* or cell culture-based effector function assays may not entirely correspond to the contributions of S309-GA-AFUC to ADCP activity *in vivo*. Our flow cytometry profiling, ventilation, and transcriptomic analyses suggest that monocytes and resident interstitial macrophages contribute to the enhanced protection of S309-GA-AFUC by more rapidly mitigating SARS-CoV-2-induced inflammation and promoting lung repair compared to parental S309-treated animals.

Growing evidence demonstrates the importance of Fc-FcγR interactions in antibody protection against viruses. Greater *in vitro* Fc effector functional activity of antibodies correlates with a lower risk of developing severe acute disease or long-term sequelae after infection with EBOV, influenza virus, type 1 human immunodeficiency virus (HIV-1), and SARS-CoV-2 (29, 46–51). In models of arthritogenic alphavirus infection, non-neutralizing anti-E1 antibodies require Fc-FcγR engagement to confer protection against viral burden and disease (52–54). Similar Fc-dependent mechanisms were observed with mAbs against influenza A virus, where the protection against morbidity and mortality conferred by broadly reactive but non-neutralizing antibodies required Fc-FcγR interactions (23, 55).

Several studies have shown that the *in vitro* neutralizing potency of some anti-SARS-CoV-2 antibodies does not directly correlate with *in vivo* efficacy (6, 21, 22, 45). Potential explanations for this discrepancy lie in the heterogeneity of epitope specificity and the ability to mediate Fc effector function activities. Studies with mAbs against SARS-CoV-2 demonstrated epitope-dependent accessibility of the spike protein on the surface of cells, with greater binding of antibodies to the N-terminal domain (NTD), S2 subunit, and a subset of RBD epitopes (20, 56, 57). Binding kinetics, along with the geometry of engagement, could differentially impact the ability of antibodies to promote certain Fc effector functions (18, 23). Beyond this, the strong immune selective pressure has resulted in the emergence of variants with substitutions that disrupt or abrogate the neutralization capacity of many anti-RBD antibodies (58).

The importance of Fc effector functions is consistent with clinical observations in which vaccines targeting historical SARS-CoV-2 variants continue to protect against severe disease caused by antigenically drifted or shifted strains (59, 60), although some of this effect also might be mediated by inhibitory T cell responses (61). Protection against SARS-CoV-2 challenge in mice mediated by passive transfer of commercially available human immune globulin (IVIG) or vaccine-elicited mouse immune sera required FcγR expression, particularly FcγR III (62, 63). Similarly, the efficacy of passively transferred, poorly neutralizing convalescent sera that delayed SARS-CoV-2 lethality in K18-hACE2 mice correlated with Fc effector function activity *in vitro* (64). Related to this point, neutralizing antibodies with mutations that abrogate Fc effector functions (e.g., LALA-PG or GRLR) showed reduced potency in BALB/c and K18-hACE2 mice or hamsters (18, 19, 21, 63). In contrast, vaccine-elicited non-neutralizing antibodies conferred protection in K18-hACE2 and hamsters in an exclusively Fc-dependent protective manner, engaging C1q and mediating ADCC *in vitro* (65). Indeed, despite the markedly reduced *in vitro* neutralization of some Omicron subvariants, S309 retained the ability to inhibit infection in mice and mediate Fc effector function activities (6, 7). These studies, along with our current experiments, highlight the contribution of Fc-mediated effector functions to antibody therapeutics and provide a rationale for Fc-engineering strategies to enhance antiviral efficacy.

A panel of Fc-modified variants of S309 exhibited distinct FcγR binding profiles and *in vitro* effector function activities. These included GRLR, GA, AFUC, and the combined GA-AFUC variant. S309-GA-AFUC induced greater *in vitro* ADNP activity than parental S309, demonstrating the potential impact of Fc modifications on effector functions. While parental S309 reduced SARS-CoV-2 viral burden in both the upper and lower airways of Hu-FcγR Tg mice compared to isotype control, the S309-GA-AFUC variant conferred greater protection. Our results are consistent with studies that optimized antibody protection against mouse-adapted SARS-CoV-2 using Fc mutations S239D/A330L/I332E and GA and ALIE, which exhibit increased binding to FcγRs IIA and IIIA (22, 66).

Monocytes and macrophages are important cell types for mediating Fc-dependent control of viral burden, inflammation, and lethality (21, 52, 53, 67). We observed greater inhibitory effects of S309-GA-AFUC at early time points (2 or 4 dpi), which likely precede effects on adaptive immunity and point to FcγR-triggered innate immune-dependent viral clearance mechanisms. Indeed, inhibition of the trafficking of CCR2-expressing cells reduced the protective effect of S309-GA-AFUC in the lung. CCR2^+^ cells (principally monocytes) are mobilized from the bone marrow in response to inflammation, infiltrate the lung, and differentiate into monocyte-derived interstitial, alveolar, and conventional macrophages (68, 69). As CCR2^+^ myeloid cells express multiple activating FcγRs, they may mediate enhanced viral clearance by more effectively engaging with S309-GA-AFUC and phagocytosing infected cells and opsonized virions. Our flow cytometry and transcriptomic analyses also suggest that S309-GA-AFUC treatment better limits pro-inflammatory activation of resident interstitial macrophages and classical monocytes after SARS-CoV-2 infection. Treatment with S309-GA-AFUC reduced CD86/CD80 expression and shifted transcriptional programs from glycolytic, stress-associated profiles observed with parental S309 treatment to oxidative, reparative states. Our results demonstrating a protective role for S309-GA-AFUC antibody in the context of CCR2-expressing cells at early time points after SARS-CoV-2 infection contrast with influenza virus experiments in the same Hu-FcγR Tg mice, which showed that CD8^+^ T cells were required for Fc-optimized antibodies to confer protection at a later time point (10 dpi) (25).

### Limitations of the study

We note several limitations in our study. (i) The use of human antibodies in transgenic mice expressing human type I FcγRs under their endogenous regulatory elements likely has greater translational relevance than studying human or mouse antibodies in conventional mice (70). Nonetheless, there may be species-dependent differences, such as the distribution and subsets of FcγR-expressing myeloid cells in the lung in the context of SARS-CoV-2 infection and the absence of human type II FcγRs in these mice (16). Type II FcγRs can have anti-inflammatory signaling functions that shape immune dynamics and pathology during lung infection. (ii) The SARS-CoV-2 B.1.351 natural isolate showed limited pathogenicity in Hu-FcγR Tg mice, necessitating the use of a mouse-adapted (MA30) virus (41) to assess the effect of S309 mAbs on lung function outcomes. Nonetheless, and despite causing quantitative defects in ventilatory function, MA30 caused less lung pathology in the Hu-FcγR Tg C57BL/6 mice than that reported in BALB/c mice or K18-hACE2 transgenic mice. (iii) The reduced activation and inflammatory states of myeloid cell subsets in the lungs of S309-GA-AFUC-treated HuFcγR Tg mice could reflect the lower viral burden. Additional kinetic analyses at earlier time points could provide insights into how FcγR engagement modulates the inflammatory environment prior to changes in viral infection. (iv) We tested a limited number of Fc variants in our study. As many other Fc substitutions have been described (71), some might have superior protective activity against SARS-CoV-2 when introduced into the S309 mAb.

In summary, enhancing Fc-FcγR interactions by mutation and glycan modification promoted viral clearance *in vivo*, which mitigated inflammation and preserved lung function. Optimal protection by S309-GA-AFUC required CCR2-expressing cells, suggesting that monocytes or monocyte-derived cell populations engage antibody-bound virions or infected cells to facilitate more efficient clearance or stimulate an antiviral and tissue reparative state through downstream signaling events. Our study expands on work defining the effects and mechanistic basis of protection of Fc-optimized antiviral antibodies. Such information may be valuable for designing therapeutic antibodies against viral variants that escape neutralization through antigenic drift.

## MATERIALS AND METHODS

### Cells

Vero-TMPRSS2 (72) and Vero-hACE2-TMPRSS2 (73) cells were cultured at 37°C in Dulbecco’s modified Eagle medium (DMEM) supplemented with 10% fetal bovine serum (FBS), 10 mM HEPES (pH 7.3), 1 mM sodium pyruvate, 1× non-essential amino acids, and 100 U/mL of penicillin–streptomycin. Vero-TMPRSS2 and Vero-hACE2-TMPRSS2 cells were supplemented with 5 μg/mL of blasticidin and 10 μg/mL of puromycin, respectively. All cells routinely tested negative for mycoplasma using a PCR-based assay.

### Viruses

All SARS-CoV-2 strains used (B.1.351 and mouse-adapted MA30) have been previously described (73, 74). Viruses were subjected to next-generation deep sequencing to confirm the presence and stability of previously established substitutions.

### FcγR binding and activation

Serial dilutions of mAbs were incubated for 25 min with Expi-CHO cells (Invitrogen) stably transduced using a lentivirus vector for expression of SARS-CoV-2 Wuhan-1 spike protein. Jurkat reporter effector cells (Promega) stably expressing FcγR IIA receptor (H131 variant) or FcγR IIIA receptor (V158 variant) on their surface and an NFAT-driven luciferase gene were added at an effector to target cell ratio of 5:1 (FcγR IIA) or 6:1 (FcγR IIIA). NFAT-driven luminescence was measured after 20 h of incubation at 37°C with 5% CO_2_ with a Biotek Synergy 2 microplate reader using the Bio-Glo-TM Luciferase Assay Reagent according to the manufacturer’s instructions (Promega, Cat. no.: G7018 and G9995).

MAbs were analyzed by a customized Luminex multiplex platform to quantify B.1.351 spike-specific FcγR-binding profiles, as previously described (75, 76). Briefly, individual B.1.351 spike antigens were covalently linked to carboxylated Magplex beads by carbodiimide-NHS ester coupling (Thermo Fisher). The antigen-coupled microspheres were washed and pooled in 0.1% BSA in PBS and then incubated with serial dilutions (0.1–100 μg/mL) of antibodies in duplicate overnight at 4°C on a shaker at 700 rpm (Greiner Bio-One). Unbound antibodies were washed away using the magnetic 384-well HydroSpeed Plate Washer (Tecan) using 1× Luminex assay buffer (1× PBS, 0.1% BSA, and 0.05% Tween-20). SA-PE-conjugated to biotinylated recombinant Fc receptors were added to each well and incubated for 1 h at room temperature with continuous shaking. Unbound complexes were washed away using the magnetic 384-well HydroSpeed Plate Washer. Beads were resuspended in 40 µL of QSOL buffer (Sartorius) and then run on the iQue Screener PLUS (Intellicyt). The results were reported as mean fluorescence intensity (MFI) to indicate relative Fc receptor binding.

### Fc effector functions

To assess ADNP, biotinylated B.1.351 spike was conjugated to fluorescent yellow-green NeutrAvidin beads (Invitrogen), as previously described (77). In a 96-well round-bottom plate, 10 µL of antigen-coated beads were incubated with 10 µL of serially diluted antibodies in duplicate at 37°C for 2 h to allow immune complex formation. Complexes were pelleted at 2,000 × *g* for 10 min, and supernatants were discarded. Fresh whole blood from healthy donors was collected in ACD tubes, mixed with ACK lysis buffer (1:10), and incubated at room temperature for 5 min with periodic inversion to lyse erythrocytes. Samples were centrifuged (1,500 rpm, 5 min), washed with PBS, and resuspended in complete RPMI supplemented with 10% FBS (R10 media), 10 mM HEPES, and 2 mM L-glutamine. Leukocytes (50,000 cells per well) were added to the pre-formed immune complexes and incubated for 1 h at 37°C, 5% CO₂. After incubation, cells were washed, stained with anti-human CD66b-Pacific Blue, fixed, and resuspended in PBS. Fluorescence was acquired on the Intellicyt iQue, and phagocytic scores were determined specifically within the CD66b^+^ neutrophil population.

To evaluate ADCD, individual B.1.351 spike antigens were covalently linked to carboxylated Magplex beads by carbodiimide-NHS ester coupling (Thermo Fisher). In a 384-well plate, 5 µL of diluted antibody was added to antigen-coupled beads in duplicate and incubated at room temperature with shaking (700 rpm) for 2 h to form immune complexes. Beads were washed and then incubated with guinea pig complement (Cedarlane) diluted in gelatin veronal buffer containing Ca²^+^ and Mg²^+^ (Boston BioProducts) for 20 min at room temperature with shaking. After washing twice with PBS + 15 mM EDTA, beads were incubated with fluorescein-labeled goat anti-guinea pig C3 (MP Biomedicals) for 30 min at room temperature. Beads were then washed with 0.1% BSA-PBS and resuspended in PBS. Fluorescence data were collected on the Intellicyt iQue and reported as MFI.

To evaluate NK cell activation, ADNKA was performed as previously described (78); 96-well round-bottom plates were coated overnight at 4°C with B.1.351 spike at 1 μg/mL, blocked with 5% bovine serum albumin in PBS, then incubated for 2 h at 37°C with 50 μL of diluted antibodies to allow immune complex formation. Primary NK cells were isolated from human buffy coats using RosetteSep (StemCell) and rested overnight in R10 media supplemented with IL-15 at 1 ng/mL. The following day, NK cells (5 × 10^4^ cells/well) were added to the antibody-coated plates in the presence of CD107a (PE-Cy5), Brefeldin A, and GolgiStop. After 5 h at 37°C, the cells were harvested, surface stained for CD3 (0.25 μL/well), CD16 (1 μL/well), and CD56 (1 μL/well) and then fixed/permeabilized using Perm A/B (Thermo). Data were analyzed within the CD3^-^CD56^+^CD16^+^ NK cell population. Results are reported as the percent of NK cells positive for CD107a.

To evaluate ADCP, biotinylated B.1.351 spike was conjugated to fluorescent yellow-green NeutrAvidin beads (Invitrogen), as previously described (77). In a 96-well round-bottom plate, 10 µL of antigen-coated beads was incubated with 10 µL of serially diluted antibodies in duplicate at 37°C for 2 h to allow immune complex formation. Complexes were pelleted at 2,000 × *g* for 10 min, and supernatants were discarded. THP-1 monocytes (25,000 cells/well) were added and incubated overnight (16 h) at 37°C in 5% CO₂. After incubation, cells were washed, fixed with 4% paraformaldehyde, and resuspended in PBS. Phagocytosis was quantified on the Intellicyt iQue. Phagocytic scores were calculated as: (%FITC^+^ cells × geometric mean fluorescence intensity of FITC^+^ cells) ÷ 10,000.

### MAb preparation

MAbs were prepared according to one of two protocols: (i) S309 was isolated from Epstein-Barr virus-immortalized memory B cells from a convalescent SARS-CoV patient (1). The S309 heavy chain Fc variants G236A (GA) and G326R/L328R (GRLR) were engineered by site-directed mutagenesis using overlap extension PCR. S309 (parental), S309-GRLR, and S309-GA plasmids (1:1 ratio of heavy and light chains) were transfected into Expi-CHO cells, as previously described (79). To produce S309-AFUC and S309-GA-AFUC variants, 100 µM 2-fluoro-L-fucose (MedChemExpress) was added to the medium of cells transfected with S309 or S309-GA plasmids, respectively. Antibodies were affinity-purified using HiTrap Protein A columns and sterilized via passage through a 0.22-µm filter. (ii) S309-GRLR and S309 plasmids (1:1 ratio of heavy and light chains) were diluted into Opti-MEM, complexed with ExpiFectamine 293 transfection reagent (Thermo Fisher), transfected into Expi293 cells (Thermo Fisher), and cultured at 37°C. Transfected cells were supplemented 1 day later with Expi 293 transfection enhancer 1 and 2. S309-GA plasmids (1:1 ratio of heavy and light chains) were diluted into Opti-MEM, mixed with P3000 reagent, complexed with lipofectamine 3000 diluted in Opti-MEM, then transfected into HEK293/FUT8 KO cells (80), and cultured at 37°C. Transfected cells were supplemented with DMEM supplemented with 10% FBS (ultra-low IgG, Thermo Fisher), 10 mM HEPES pH 7.3, 1 mM sodium pyruvate, 1× non-essential amino acids, and 100 U/mL of penicillin–streptomycin. One day post-transfection, media were replaced with fresh, complete DMEM. Supernatants were harvested 4 (S309-GA) or 5 (S309-GRLR and S309) days after transfection, centrifuged at 3,000 × *g* to remove cell debris, purified using Protein A Sepharose 4B (Thermo Fisher), and dialyzed into PBS. 

### ELISA

Recombinant WA1/2020 spike protein (2 μg/mL) was immobilized on 96-well Maxisorp ELISA plates (Thermo Fisher) overnight at 4°C in coating buffer (1× PBS supplemented with 0.05% Tween-20, 2% BSA, and 0.02% NaN_3_). Plates were washed with PBS and blocked with 4% BSA for 1 h at 25°C. MAb was serially diluted in 2% BSA and incubated on plates for 1 h at 25°C. After washing with PBS, spike-bound antibodies in serum were detected with horseradish peroxidase (HRP)-conjugated goat anti-human IgG at a dilution of 1:1,000 (Leinco, Cat. no. H605, RRID: AB_2892915) and incubated for 2 h at 25°C. Plates were washed and developed with the 3,3′,5,5′-tetramethylbenzidine substrate (Thermo Fisher), and the reaction was stopped with 2 N H_2_SO_4_ and read at 450 nm using a microplate reader (BioTek).

### Focus reduction neutralization assay

Serial dilutions of mAbs were incubated with 10^2^ FFU of B.1.351 or MA30 for 1 h at 37°C. Antibody-virus complexes were added to Vero-TMPRSS2 cell monolayers in 96-well plates and incubated at 37°C for 1 h. Subsequently, cells were overlaid with 1% (wt/vol) methylcellulose in MEM. Plates were collected 42 h (B.1.351) or 30 h (MA30) later by removing overlays and fixed with 4% paraformaldehyde (PFA) in PBS for 20 min at room temperature. Plates were washed and sequentially incubated with an oligoclonal pool (SARS2-02, -08, -09, -10, -11, -13, -14, -17, -20, -26, -27, -28, -31, -38, -41, -42, -44, -49, -57, -62, -64, -65, -67, and −71) (81) of anti-spike murine antibodies at a dilution of 1:5,000 and HRP-conjugated goat anti-mouse IgG at a dilution of 1:500 (Thermo Fisher, Cat. no. 31430) in PBS supplemented with 0.1% saponin and 0.1% bovine serum albumin. SARS-CoV-2-infected cell foci were visualized using TrueBlue peroxidase substrate (KPL) and quantitated on an ImmunoSpot microanalyzer (Cellular Technologies).

### Mouse experiments

Virus inoculations were performed under anesthesia that was induced and maintained with ketamine hydrochloride and xylazine, and all efforts were made to minimize animal suffering. No sample size calculations were performed to power each study. Sample size for animal experiments was determined based on criteria set by the institutional Animal Care and Use Committee and prior virus challenge experiments. Data distribution was assumed to be normal, although this was not formally tested. Experiments were neither randomized nor blinded, and mice were randomly assigned to treatment groups.

Hu-FcγR Tg mice expressing all human FcγRs on the FcRα-null C57BL/6J genetic background were a generous gift (J. Ravetch, Rockefeller University), bred at Washington University, and have been described previously (16). Heterozygous K18-hACE C57BL/6J mice [strain: 2B6.Cg-Tg(K18-ACE2)2Prlmn/J] were obtained from The Jackson Laboratory. Male mice were housed in groups of four to five; photoperiod, 12 h on:12 h off dark/light cycle. Ambient animal room temperature was at 70°F, controlled within ±2°F, and room humidity was 50%, controlled within ±5%. Animals were fed standard chow diets and monitored daily.

For B.1.351 challenge experiments, 12-week-old Hu-FcγR Tg male mice were administered S309-GRLR, S309, S309-GA, S309-AFUC, S309-GA-AFUC, or isotype control (3 or 10 mg/kg) by intraperitoneal injection either 1 day before or 8 h after intranasal infection with 1 × 10^5^ FFU of SARS-CoV-2 B.1.351 in a final volume of 50 μL. Lungs, nasal turbinates, and nasal washes (collected in 1 mL of 0.5% bovine serum albumin in PBS) were collected at 2 (prophylaxis studies) or 4 (therapeutic studies) dpi for virological analysis.

For MA30 challenge experiments, 24-week-old Hu-FcγR Tg male mice were administered different doses of S309, S309-GA-AFUC, or isotype control by intraperitoneal injection, depending on the experimental question. To assess differences in virological protection, mice were administered 6 mg/kg of antibody at −24 h and then harvested at 4 dpi. The antibody dose was increased to account for the enhanced viral infection in the MA30 model compared to B.1.351. For evaluation of lung ventilatory function and histopathology, mice were administered a lower 1 mg/kg dose of antibody at −24 h and then harvested at 7 dpi, which facilitated differences in disease progression between treatment groups. At day 0 (1 day after mAb treatment), mice were inoculated intranasally with 6 × 10^5^ FFU of SARS-CoV-2 MA30 in a final volume of 50 μL. Lungs, nasal turbinates, and nasal washes (collected in 1 mL of 0.5% bovine serum albumin in PBS) were collected at 4 dpi for virological and flow cytometric analyses.

### Measurement of viral RNA

Tissues were weighed and homogenized with zirconia beads in a MagNA Lyser instrument (Roche Life Science) in 1 mL of DMEM supplemented with 2% heat-inactivated FBS. Tissue homogenates were clarified by centrifugation at 10,000 × *g* for 5 min and stored at −80°C. RNA was extracted using the MagMax mirVana Total RNA isolation kit (Thermo Fisher Scientific) on the Kingfisher Flex extraction robot (Thermo Fisher Scientific). RNA was reverse transcribed and amplified using the TaqMan RNA-to-CT 1-Step Kit (Thermo Fisher Scientific). Reverse transcription was carried out at 48°C for 15 min, followed by 2 min at 95°C. Amplification was accomplished over 50 cycles as follows: 95°C for 15 s and 60°C for 1 min. Copies of SARS-CoV-2 *N* gene genomic RNA in samples were determined using a published assay (82).

### Plaque assay for infectious virus

Lung and nasal turbinate homogenates were serially diluted and added to Vero-hACE2-TMPRSS2 cell monolayers in 24-well tissue culture plates for 1 h at 37°C. Cells were then overlaid with 1% (wt/vol) methylcellulose in MEM and incubated for 72 h (B.1.351) or 96 h (MA30). Subsequently, cells were fixed with 4% PFA in PBS for 20 min at room temperature before staining with 0.05% (wt/vol) crystal violet in 20% methanol. Viral plaques were counted manually.

### Blockade of monocyte recruitment

To inhibit monocyte trafficking and recruitment, anti-CCR2 (clone MC-21; 50 μg) (39) or an isotype control mAb (BioXCell; clone LTF-2; 50 μg) was administered to mice by intraperitoneal injection 1 day before and after SARS-CoV-2 B.1.351 inoculation. Lungs, nasal turbinates, and nasal washes (collected in 1 mL of 0.5% bovine serum albumin in PBS) were collected at 2 dpi for virological analysis. Anti-CCR2 or an isotype control mAb was administered to mice by intraperitoneal injection 1 day before, at D+1, and D+3 relative to SARS-CoV-2 MA30 inoculation. Lungs, nasal turbinates, and nasal washes (collected in 1 mL of 0.5% bovine serum albumin in PBS) were collected at 4 dpi for virological analysis.

### Neutrophil depletion

Anti-Ly6G (BioXCell; clone 1A8-CP129) or an isotype control (BioXCell; clone C1.18.4) was administered to mice by intraperitoneal injection at D-1, D+1, and D+3 relative to SARS-CoV-2 MA30 inoculation. Lungs, nasal turbinates, and nasal washes (collected in 1 mL of 0.5% bovine serum albumin in PBS) were collected at 4 dpi for virological and immunological analyses.

### Respiratory mechanics

Seven days following the MA30 challenge, mice were anesthetized with ketamine/xylazine (100 and 10 mg/kg intraperitoneally, respectively). The trachea was isolated via dissection of the neck area and cannulated using an 18-guage blunt metal cannula (typical resistance of 0.18 cm H_2_O s/mL), which was secured in place with a nylon suture. The mouse was then connected to the flexiVent computer-controlled piston ventilator (SCIREQ) via the cannula, which was attached to the FX adaptor Y-tubing. Mechanical ventilation was initiated, and mice were given an additional 100 mg/kg of ketamine and 0.1 mg per mouse of the paralytic pancuronium bromide via the intraperitoneal route to prevent breathing against the ventilator and during measurements. Mice were ventilated using default settings for mice, which consisted of a positive end-expiratory pressure at 3 cm H_2_O, a 10 mL/kg tidal volume, a respiratory rate at 150 breaths per minute, and a fraction of inspired oxygen of 0.21 (i.e., room air). Respiratory mechanics were assessed using the forced oscillation technique, as previously described (83), using the latest version of the flexiVent operating software (flexiWare version 8.1.3). Pressure–volume loops and measurements of inspiratory capacity were also done.

### Histology

Four days after the MA30 challenge, mice were euthanized immediately before harvest and fixation of tissues. The lungs were inflated with ~2  mL of 4% PFA using a 3-mL syringe and a catheter inserted into the trachea. For fixation after infection, organs were kept in a 40-mL suspension of 4% PFA for 1 day before processing. Tissues were embedded in paraffin and sections were stained with hematoxylin and eosin. An uninfected mouse was used as a negative control and stained in parallel. Tissue sections were visualized using a 3DHISTECH Pannoramic P250 Flash III.

### Flow cytometry analysis

Four days following MA30 infection, mice were anesthetized with ketamine/xylazine (100 and 10 mg/kg via intraperitoneal injection, respectively). After mice were anesthetized, 100 μL of CD45.2 antibody (Pacific Blue; BioLegend) was administered at a 1:10 dilution in PBS by retro-orbital injection to stain intravascular cells. Two minutes after CD45.2 mAb injection, mice were euthanized and then perfused with sterile PBS, and the left lobe was collected for virological analysis. The four right lobes were digested in 50 μL of 5 mg/mL of Liberase Tm (Roche) and 12.5 μL of 10 mg/mL of DNase I (Sigma-Aldrich) in 5 mL of HBSS at 37°C for 35 min. Single-cell suspensions of lung digest were pre-incubated with Fc block antibody (BioLegend) in PBS + 2% heat-inactivated FBS + 1  mM EDTA for 20 min at 4°C. Cells were then stained with antibodies against CD45 (AF700; clone 30-F11; BioLegend), CD88 (BV605; BD Biosciences), CD11b (BV650; clone M1/70; BioLegend), CD11c (APC-Cy7; clone N418; BioLegend), CD172a (APC; clone P84; BioLegend), F4/80 (PE-Cy5; clone BM8; BioLegend), Ly6C (PerCP/Cy5.5; clone HK1.4; BioLegend), MHC-II (BV711; clone M5; BioLegend), Siglec F (PE-Dazzle 594; clone S17007L; BioLegend), XCR1 (BV785; clone ZET; BioLegend), CD40 (FITC; clone 3/23; BioLegend), CD80 (BV421; clone 16-10A1; BioLegend), CD86 (PE-Cy7; clone GL-1; BioLegend), CD36 (PE-Cy7; clone HM36; BioLegend), CX3CR1 (BV785; clone SA011F11; BioLegend), CD209 (APC; clone MMD3; BioLegend), CD80 (BV650; clone 16-10A1; BioLegend), CD86 (PE-CF594; clone GL1; BD Biosciences), CD209 (BV421; clone MMD3; BioLegend), CD80 (APC; clone 16-10A1; BioLegend), Ly6G (Spark Blue 550; clone 1A8; BioLegend), NK1.1 (Biotin; clone PK136, ThermoFisher), Ly6G (Biotin; clone 1A8; BioLegend), Siglec F (Biotin; clone S17001L; BioLegend), TCRβ (Biotin; clone H57-597; BioLegend), CD19 (Biotin; clone 6D5; BioLegend), streptavidin (PE; BioLegend), fixable viability dye (Live/Dead aqua), True-Stain Monocyte Blocker (5 μL per sample), and Brilliant Stain Buffer Plus (10 μL per sample) and then fixed with 4% PFA in PBS for 20 min at room temperature. All antibodies were used at a dilution of 1:200, with the exceptions of MHC-II BV711, CD88 BV605, CD80 BV421, CD86 PE-Cy7, CD36 PE-Cy7, CD80 APC, and CD86 PE-CF594, which were used at 1:100, and the viability dye, which was used at 1:2,000. Absolute cell counts were determined using Precision Count Beads (BioLegend). Flow cytometry data were acquired on a 3-laser Cytek Aurora cytometer (CytekBio) and analyzed using FlowJo software (version 10.10.0).

### Sorting of myeloid cells for bulk RNA sequencing

Three days after MA30 infection, mice were anesthetized with ketamine/xylazine (100 and 10 mg/kg via intraperitoneal injection, respectively), and 100 μL of CD45.2 antibody (APC; clone 104; BioLegend) was administered at a 1:10 dilution in PBS by retroorbital injection to identify intravascular cells. Two minutes later, mice were euthanized and then perfused with sterile PBS. Lungs were digested in 50 μL of Liberase Tm (5 mg/mL, Roche) and 12.5 μL of DNase I (10 mg/mL, Sigma-Aldrich) in 5 mL of HBSS at 37°C for 35 min. Single-cell suspensions were pre-incubated with Fc block antibody (BioLegend) in PBS + 2% heat-inactivated FBS + 1  mM EDTA for 20 min at 4°C. Cells were then divided into two groups for sorting. For interstitial macrophages, cells were stained with antibodies against CD45 (PE-Cy7; clone 30-F11; BioLegend), MHC-II (BV421; clone M5; BioLegend), CD88 (PE; clone 20/70; BioLegend), and CX3CR1 (BV510; clone SA011F11; BioLegend). For classical monocytes, cells were stained with antibodies against CD45 (PE-Cy7; clone 30-F11; BioLegend), Ly6G (BV510; clone 1A8; BioLegend), Ly6C (PE; clone HK1.4; BioLegend), and CD115 (BV421; clone AFS98; BioLegend). All samples were stained with fixable viability dye (zombie green), True-Stain Monocyte Blocker (5 μL per sample), and Brilliant Stain Buffer Plus (10 μL per sample). All antibodies were used at a dilution of 1:200, with the exceptions of MHC-II BV421, CD88 PE, and Ly6C PE, which were used at 1:100, and the viability dye, which was used at 1:2,000. Following incubation in PBS + 2% heat-inactivated FBS + 1  mM EDTA for 20 min at 4°C, cells were sorted on a MACSQuant Tyto instrument (Miltenyi Biotec).

### RNA sequencing

Following cell sorting, RNA was extracted using the Arcturus PicoPure RNA Isolation Kit (Thermo Fisher Scientific) according to the manufacturer’s instructions. RNA purity was confirmed using a NanoPhotometer spectrophotometer (IMPLEN). RNA concentration and quality were assessed using the RNA Nano 6000 Assay Kit of the Bioanalyzer 2100 system (Agilent Technologies). One microgram of RNA per sample was used as input material for the RNA sample preparations. Sequencing libraries were generated using NEBNext UltraTM RNA Library Prep Kit for Illumina (NEB, USA) following the manufacturer’s recommendations, and index codes were added to attribute sequences to each sample. Briefly, mRNA was purified from total RNA using poly-T oligo-attached magnetic beads. Fragmentation was carried out using divalent cations under elevated temperature in NEBNext First Strand Synthesis Reaction Buffer (5×). First-strand cDNA was synthesized using a random hexamer primer and M-MuLV Reverse Transcriptase (RNase H-). Second-strand cDNA synthesis was performed using DNA Polymerase I and RNase H. Remaining overhangs were converted into blunt ends via exonuclease/polymerase activities. After adenylation of the 3′ ends of DNA fragments, NEBNext Adaptor with hairpin loop structure was ligated to prepare for hybridization. To select cDNA fragments of 150–200 bp in length, the library fragments were purified with the AMPure XP system (Beckman Coulter, Beverly, USA). Then, 3 µL of USER Enzyme (NEB) was added with size-selected, adaptor-ligated cDNA at 37°C for 15 min, followed by 5 min at 95°C before PCR with Phusion High-Fidelity DNA polymerase, Universal PCR primers, and an Index Primer. Finally, PCR products were purified (AMPure XP system), and the library quality was assessed on an Agilent Bioanalyzer 2100 system. The clustering of the index-coded samples was performed on a cBot Cluster Generation System using PE Cluster Kit cBot-HS (Illumina) according to the manufacturer’s instructions. After cluster generation, the library preparations were sequenced on an Illumina platform, and paired-end reads were generated.

### RNA sequencing analysis

RNA-seq reads were aligned to the *Mus musculus* reference genome (GRCm39) using HISAT2 (v2.2.1). Gene-level quantification was performed with featureCounts from the Subread package (v2.0.6) using Ensembl annotations (Mus_musculus_Ensemble_107_new). Across samples, the median mapping rate was ~94%. Raw counts were imported into R (v4.4.0) for downstream analysis.

Differential expression analysis was performed using DESeq2 (v1.44.0). For each cell type, count matrices were subset and modeled with design = infection. Size factors were estimated to normalize for library depth, dispersions were fitted using empirical-Bayes shrinkage, and Wald tests were used for inference. Log₂ fold changes were shrunk using apeglm, and significance was defined as FDR < 0.05. Variance-stabilized expression values were generated using the variance stabilizing transformation (VST) function. Principal component analysis (PCA) was performed with PCAtools (v2.16.0) for the top 12,000 most variable genes. Gene set enrichment analysis was conducted with fgsea (v1.30.0) using Wald statistics to rank genes. Gene Ontology Biological Process sets were obtained from msigdbr (v25.1.1), and pathways with FDR < 0.05 were considered significant.

Heatmaps were generated with ComplexHeatmap (v2.21.1) from variance-stabilized, gene-wise *z*-scored values. For infiltrating monocytes, pathway heatmaps included genes meeting FDR < 0.05, |log₂FC| > 2, and detection in ≥3 samples; for interstitial macrophages, all pathway genes with FDR < 0.05 were used. Dot plots summarizing enriched pathways were produced using ggplot2 (v3.5.1), displaying the top positively and negatively enriched pathways per comparison.

### Statistical analysis

All statistical tests were performed as described in the indicated figure legends using GraphPad Prism version 10.4.2. Statistical significance was assigned to *P* values < 0.05, determined using a one-way ANOVA with a post-test correction when comparing three or more groups. When comparing two groups, a Mann-Whitney test was performed. The number of independent experiments performed is indicated in the relevant figure legends.

## Data Availability

All data supporting the findings of this study are available within the paper, its supplemental material, or in the Digital Commons Data@Becker repository managed by Washington University School of Medicine (https://doi.org/10.17632/t43mw66sy5). This paper does not include original code. The RNA sequencing data are available at Gene Expression Omnibus (Project accession: GSE313869). Any additional information related to the study is available from the corresponding author upon request.

## SUPPLEMENTAL MATERIAL

Supplemental Figures mbio.00557-26-s0001.pdf Fig. S1-S8.

ASM does not own the copyrights to Supplemental Material that may be linked to, or accessed through, an article. The authors have granted ASM a non-exclusive, world-wide license to publish the Supplemental Material files. Please contact the corresponding author directly for reuse.
