# Supplementary material for: Fc-engineered antibodies enhance protection against SARS-CoV-2 lung infection and inflammation
Source: mBio. 2026 Apr 13;17(5):e00557-26. doi: 10.1128/mbio.00557-26 (PMC13170307; doi:10.1128/mbio.00557-26)
Supplement: Supplemental Figures — Fig. S1-S8. [file mbio.00557-26-s0001.pdf]

Supplemental Material

Fc-engineered antibodies enhance protection against SARS-CoV-2 lung infection and inflammation

Samantha R. Mackin[1,2], Chieh-Yu Liang[1,2], Courtney E. Karl[1,3], Maksim Kleverov[2], Mehak Z. Khan[4], Thendral Selvam[4], Matthias Mack[5], Galit Alter[4], Barbara Guarino[6], Davide Corti[6], Michael A. Schmid[6], and Michael S. Diamond[1.2,3,7,8].

[1]Department of Medicine, Washington University School of Medicine, St. Louis, MO

[2]Department of Pathology & Immunology, Washington University School of Medicine, St. Louis, MO

[3]Department of Molecular Microbiology, Washington University School of Medicine, St. Louis, MO

[4]Ragon Institute of MGH, MIT and Harvard, Cambridge, MA

[5]Department of Nephrology, University Hospital Regensburg, Regensburg, Germany

[6]Vir Biotechnology, Bellinzona, Switzerland

[7]Andrew M. and Jane M. Bursky the Center for Human Immunology and Immunotherapy Programs, Washington University School of Medicine, St. Louis, MO

[8]Center for Vaccines and Immunity to Microbial Pathogens, Washington University School of Medicine, St. Louis, MO

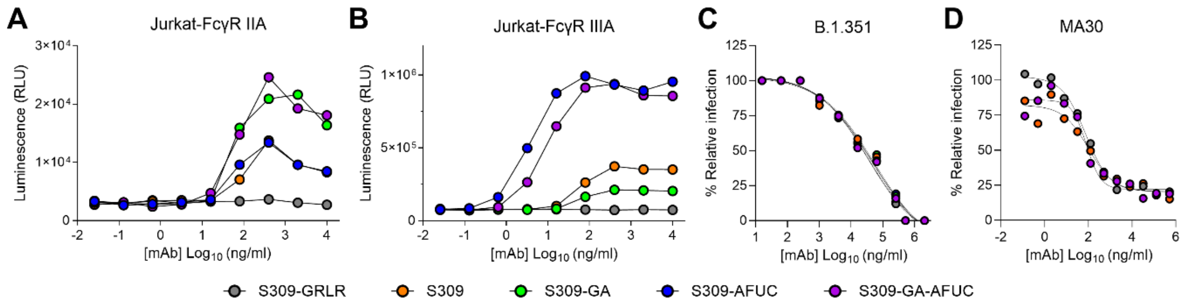

Figure S1. Fc effector function characterization of S309 Fc variants. A-B. NFAT-driven luminescence as readout for activation of FcγR IIA (**A**) or IIIA (**B**) in Jurkat cells after activation with SARS-CoV-2 Wuhan-1 spike-expressing Expi-CHO target cells opsonized with serial dilutions of S309-GRLR, S309 (parental), S309-GA, S309-AFUC, or S309-GA-AFUC (two experiments, two technical replicates). **C-D.** Neutralizing antibody responses against SARS-CoV-2 B.1.351 (**C**) or MA30 (**D**) in Vero-TMPRSS2 cells using S309-GRLR, S309 (parental), S309-GA, S309-AFUC, or S309-GA-AFUC. (one experiment, two technical replicates).

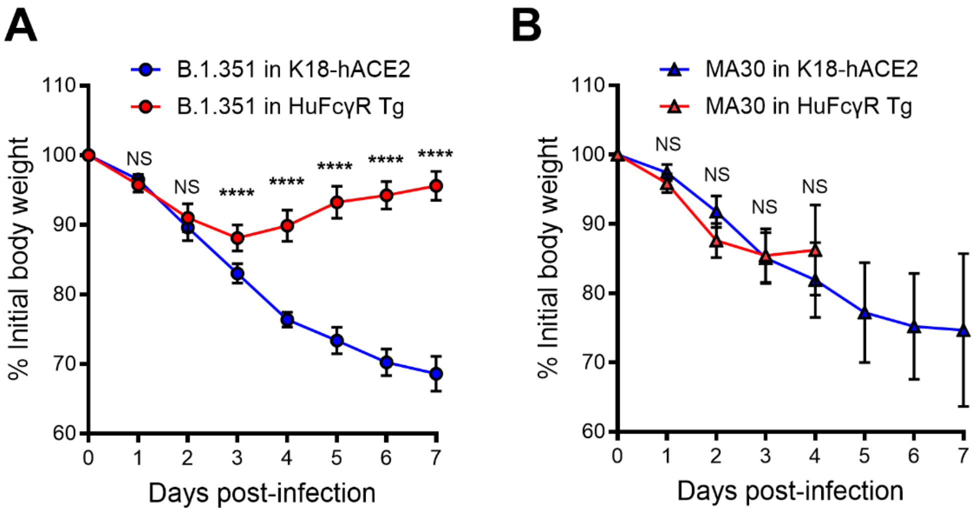

Figure S2. Weight loss following SARS-CoV-2 B.1.351 or MA30 challenge. A. Twelve-week-old male K18-hACE2 and Hu-FcγR Tg mice were inoculated by the intranasal route with 10^5 FFU of SARS-CoV-2 B.1.351 (n = 8 mice per group, two experiments). **B.** Twelve-week-old male K18-hACE2 and twenty-four-week-old male Hu-FcγR Tg mice were inoculated by the intranasal route with 6 x 10^5 FFU of MA30 (K18-hACE2 mice, n = 10; Hu-FcγR Tg mice, n = 4; one experiment). Mice were monitored for weight change (bars indicate mean ± SEM). Statistical analysis: two-way ANOVA with Sidak's post-test (NS, not significant; ****$P < 0.0001$).

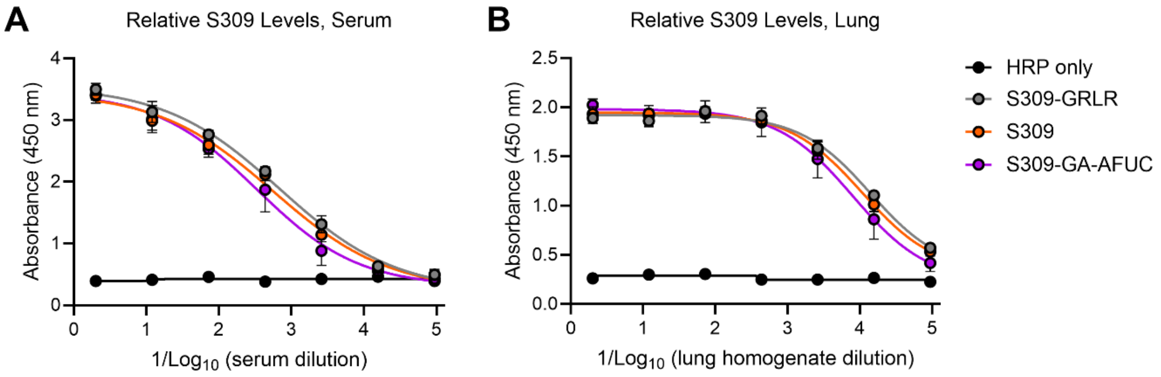

Figure S3. Levels of S309 antibody in the sera and lungs of Hu-FcγR Tg mice. Twelve-week-old male Hu-FcγR Tg mice were administered 3 mg/kg of S309-GRLR, S309, or S309-GA-AFUC by intraperitoneal injection**.** At 3 days post-administration, sera (**A**) and lungs (**B**) were collected, and levels of human anti-spike (B.1.351) IgG were determined by ELISA (*n* = 5 mice per group).

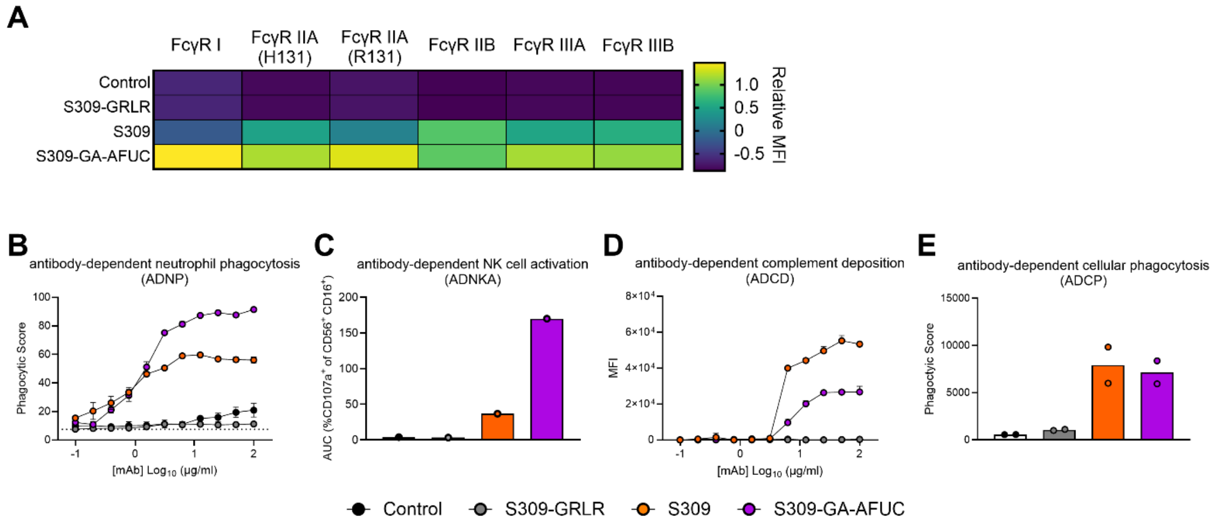

Figure S4. Fc binding and functional analysis of S309 Fc variants. A. S309-GRLR, parental S309, and S309-GA-AFUC were analyzed for SARS-CoV-2 B.1.351 spike-specific FcγR binding. Z-scored median fluorescence intensity (MFI): yellow increased, blue decreased. **B.** Antibody-mediated cellular phagocytosis with neutrophils (ADNP) using S309-GRLR, parental S309, or S309-GA-AFUC and beads coated with SARS-CoV-2 B.1.351 spike proteins (bars indicate mean ± SEM; *n* = 2 donors per group, one experiment). **C.** Antibody-dependent NK cell activation (ADNKA) using S309-GRLR, parental S309, or S309-GA-AFUC and beads coated with SARS-CoV-2 B.1.351 spike proteins (one experiment). The area under the curve (AUC) is shown for each antibody. **D.** Deposition of complement (ADCD) on beads coated with SARS-CoV-2 B.1.351 spike proteins after treatment with S309-GRLR, S309, or S309-GA-AFUC (bars indicate mean ± SEM; *n* = 2 technical replicates per group, one experiment). **E.** Antibody-mediated cellular phagocytosis with THP-1 cells (ADCP) using S309-GRLR, parental S309, or S309-GA-AFUC and beads coated with SARS-CoV-2 B.1.351 spike proteins (bars indicate mean ± SEM).

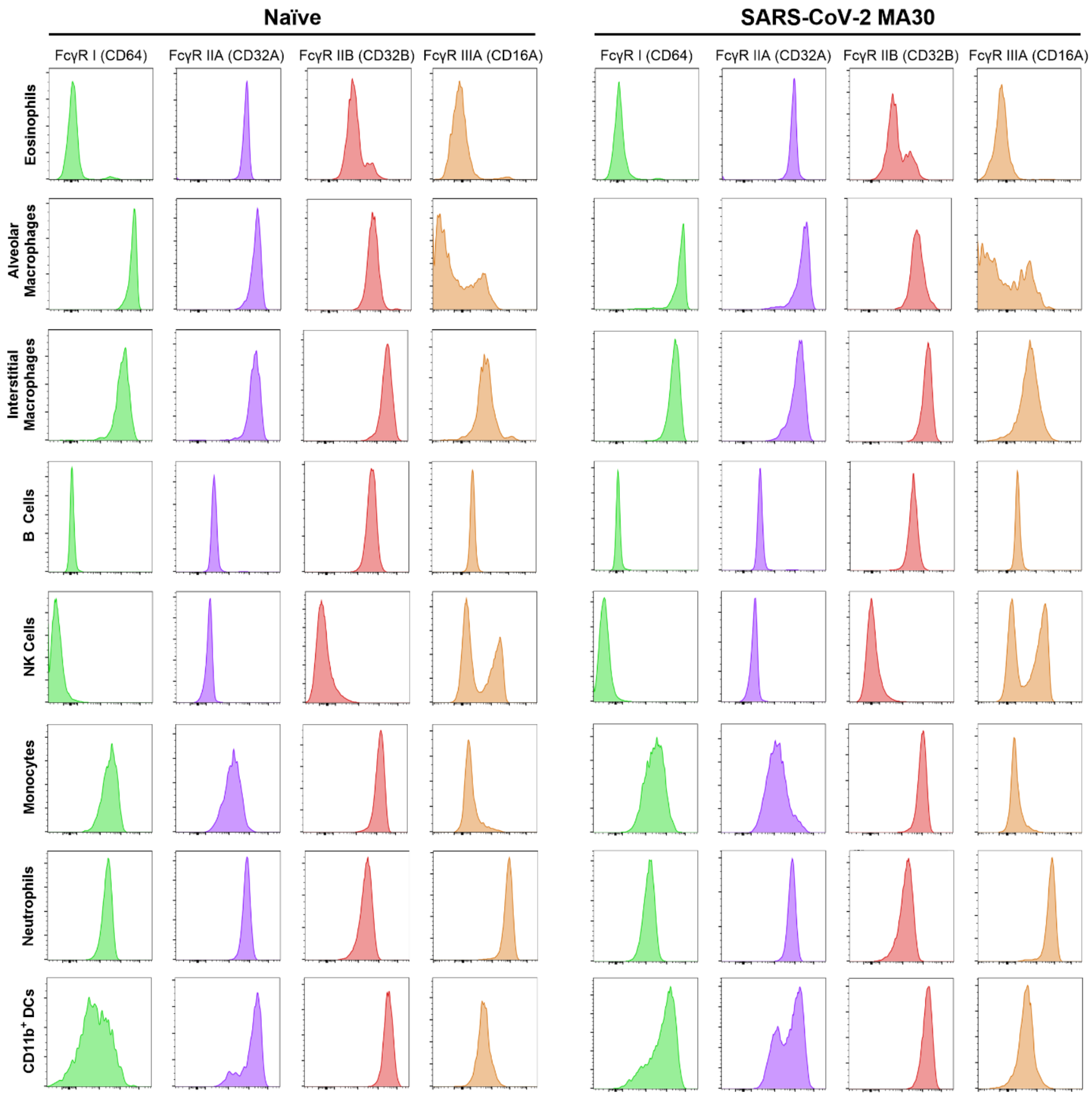

Figure S5. Expression of human FcγRs on immune cell subsets in Hu-FcγR Tg mice. Flow cytometric analysis of human FcγR expression of immune cell subsets in twelve-week-old male Hu-FcγR Tg mice, naïve or inoculated by the intranasal route with 6 x 10^5 FFU of SARS-CoV-2 MA30. Tissues were collected at 4 dpi. Data are representative of 3 mice per group.

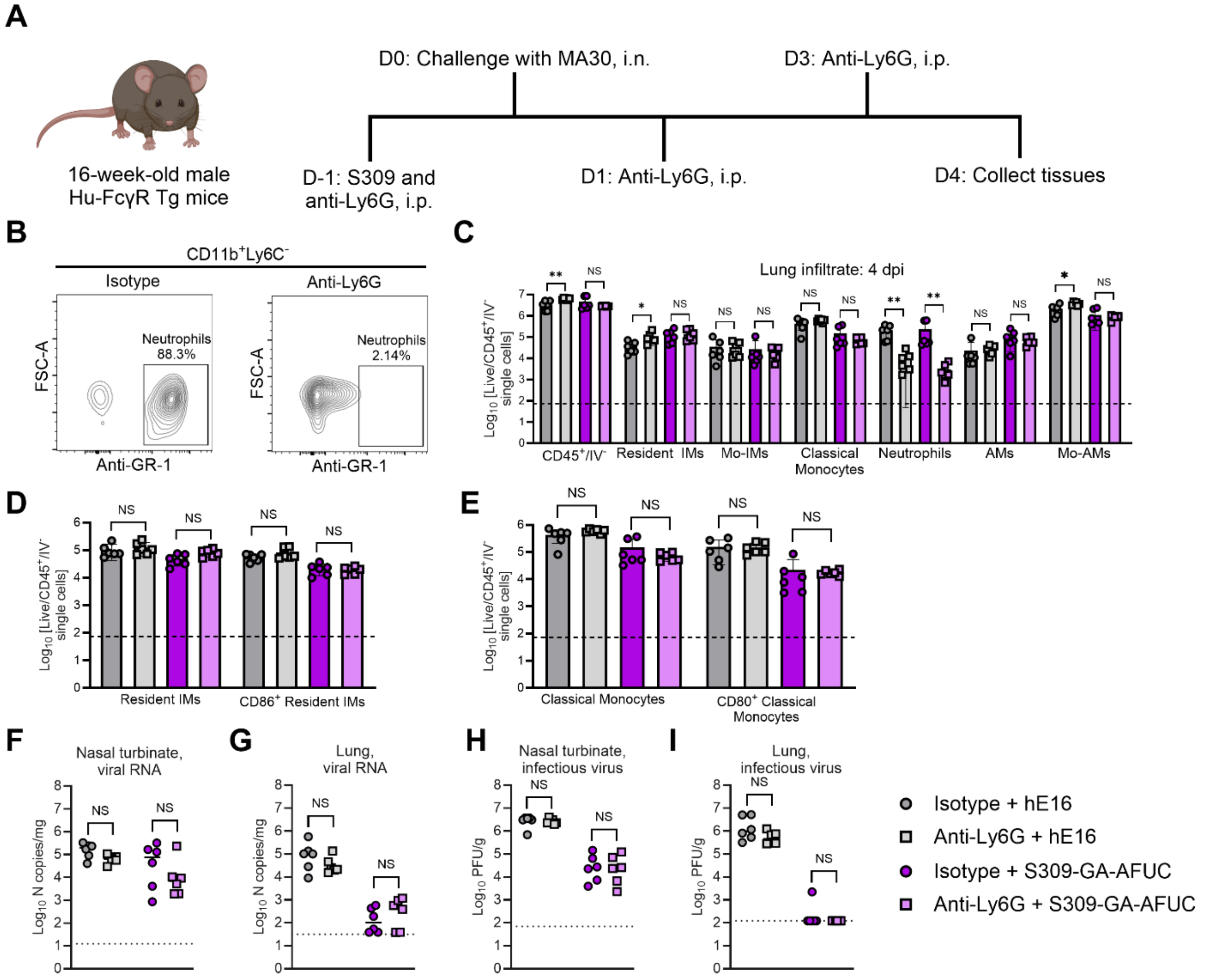

Figure S6. Anti-Ly6G treatment does not impact the protective activity of S309-GA-AFUC against MA30 infection. A. Scheme of antibody administration, virus challenge, and tissue collection. **B.** Representative flow cytometry plots showing depletion of neutrophils in the lung with numbers indicating the cell population as a percentage of CD11b^{+}Ly6C^{-} cells. **C-E.** Flow cytometric analysis of immune cell subsets in the lungs of mice receiving anti-Ly6G or isotype antibody at 4 dpi. Dotted lines show the LOD. **F-I.** Sixteen-week-old male Hu-FcγR Tg mice were administered 6 mg/kg of hE16 or S309-GA-AFUC by intraperitoneal injection on D-1, +1, and +3 relative to challenge. At 4 dpi, viral RNA in the nasal turbinates (**F**) and lungs (**G**) was quantified, and infectious virus in the turbinates (**H**) and lungs (**I**) was measured (bars indicate mean ± SEM; *n* = 6 mice per group, two experiments (**C-I**), dotted lines show LOD). Statistical analysis: between hE16 and S309-GA-AFUC; two-tailed Mann-Whitney test; NS, not significant, **P = 0.0022; *P = 0.026, **P = 0.0043, **P = 0.0022, **P = 0.0022, *P = 0.026 (**C**).

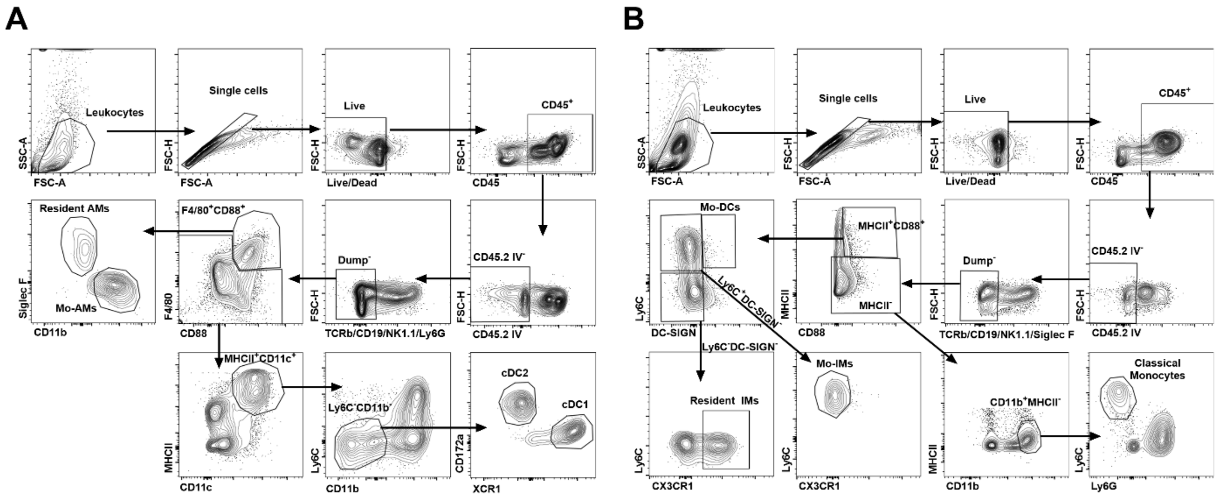

Figure S7. Flow cytometric gating strategy of lung tissues. A. Immune cell populations in the lungs of Hu-FcγR Tg mice were analyzed using the indicated gating scheme and conjugated antibodies. After gating on single, live, $CD45^+$ and CD45.2 IV^- cells, $TCR\beta^-$ $CD19^-$ $NK1.1^-$ $Ly6G^-$ cells were isolated from a dump gate. Cell populations were defined as follows: resident alveolar macrophages: $F4/80^+$ $CD88^+$ Siglec F^+ $CD11b^-$ cells; monocyte-derived alveolar macrophages: $F4/80^+$ $CD88^+$ Siglec F^- $CD11b^+$ cells. The $F4/80^-$ $CD88^-$ population was gated on $MHC\text{-}II^+$ $CD11c^+$ $CD11b^-$ $Ly6C^-$ cells and cDC1s and cD2s identified as $CD172a^-$ $XCR1^+$ and $XCR1^-$ $CD172a^+$, respectively. **B.** In a separate scheme, upon gating single, live, $CD45^+$ and CD45.2 IV^- cells, $TCR\beta^-$ $CD19^-$ $NK1.1^-$ Siglec F^- cells were isolated from a dump gate. After isolating $MHC\text{-}II^+$ $CD88^+$ cells, monocyte-derived dendritic cells were defined as $DC\text{-}SIGN^+$ $Ly6C^+$. From the $Ly6C^+$ $DC\text{-}SIGN^-$ gate, monocyte-derived interstitial macrophages were identified as $CX3CR1^-$ $Ly6C^+$ cells. Resident interstitial macrophages were defined as $Ly6C^-$ $DC\text{-}SIGN^-$ $CX3CR1^+$ cells. The $MHC\text{-}II^-$ $CD11b^+$ $Ly6C^+$ $Ly6G^-$ population was identified as classical monocytes.

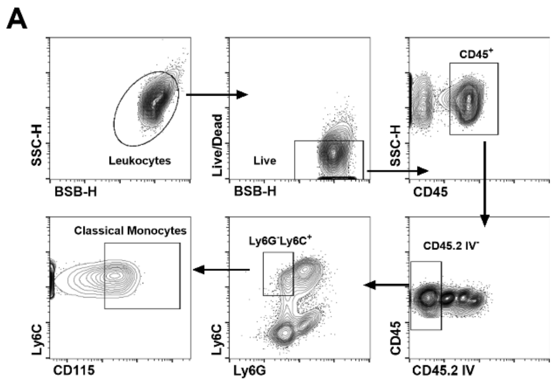

Figure S8. Flow cytometric sorting strategy of myeloid populations used for RNA sequencing. Immune cell populations in the lungs of SARS-CoV-2-infected Hu-FcγR Tg mice were analyzed at 3 dpi using the indicated gating and staining schemes. **A-B.** After gating on live, $CD45^+$ and CD45.2 IV^- cells, $Ly6G^-$ $Ly6C^+$ $CD115^+$ monocytes (**A**) and $CD88^+$ MHC-II^+ $CX3CR1^+$ interstitial macrophages (**B**) were sorted.
